# Establishment of Trophoblast‐Like Tissue Model from Human Pluripotent Stem Cells in Three‐Dimensional Culture System

**DOI:** 10.1002/advs.202100031

**Published:** 2021-11-23

**Authors:** Kangli Cui, Yujuan Zhu, Yang Shi, Tingwei Chen, Hui Wang, Yaqiong Guo, Pengwei Deng, Haitao Liu, Xiaoguang Shao, Jianhua Qin

**Affiliations:** ^1^ Division of Biotechnology Dalian Institute of Chemical Physics Chinese Academy of Sciences Dalian 116023 China; ^2^ University of Chinese Academy of Sciences Beijing 100049 China; ^3^ Dalian Municipal Women and Children's Medical Center Dalian 116037 China; ^4^ Yunnan Key Laboratory of Primate Biomedical Research Institute of Primate Translational Medicine Kunming University of Science and Technology Kunming 650031 China; ^5^ Institute for Stem Cell and Regeneration Chinese Academy of Sciences Beijing 100101 China; ^6^ CAS Center for Excellence in Brain Science and Intelligence Technology Chinese Academy of Sciences Shanghai 200031 China

**Keywords:** human pluripotent stem cells, matrices, placenta development, three‐dimensional cultures, trophoblast tissues

## Abstract

The placenta has a lifelong impact on the health of both the mother and fetus. Despite its significance, human early placental development is poorly understood due to the limited models. The models that can reflect the key features of early human placental development, especially at early gestation, are still lacking. Here, the authors report the generation of trophoblast‐like tissue model from human pluripotent stem cells (hPSCs) in three‐dimensional (3D) cultures. hPSCs efficiently self‐organize into blastocoel‐like cavities under defined conditions, which produce different trophoblast subtypes, including cytotrophoblasts (CTBs), syncytiotrophoblasts (STBs), and invasive extravillous trophoblasts (EVTs). The 3D cultures can exhibit microvilli structure and secrete human placenta‐specific hormone. Single‐cell RNA sequencing analysis further identifies the presence of major cell types of trophoblast‐like tissue as existing in vivo. The results reveal the feasibility to establish 3D trophoblast‐like tissue model from hPSCs in vitro, which is not obtained by monolayer culture. This new model system can not only facilitate to dissect the underlying mechanisms of early human placental development, but also imply its potential for study in developmental biology and gestational disorders.

## Graphical abstract

A placental trophoblast‐like tissue is generated from human pluripotent stem cells for the first time. This established tissue is composed of different trophoblast subtypes, including cytotrophoblasts (CTBs), syncytiotrophoblasts (STBs), and invasive extravillous trophoblasts (EVTs). Additionally, this tissue exhibits placental microvilli structure, thus recapitulating some essential features of human placenta at early stage.

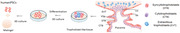

## 1 Introduction

The placenta is an extraembryonic organ that serves to provide nutrients and oxygen to the growing fetus, and plays a pivotal role in the health of both the fetus and its mother.^[^ ^1^ ^]^ In humans, the placenta is a temporary organ of fetal origin, and begins to develop upon embryo implantation into maternal endometrium.^[^ ^2^ ^]^ Upon implantation, the trophectoderm (TE) of the blastocyst proliferates rapidly and gives rise to the unique trophoblast cell subtypes located in different locations and executes distinct functions of placenta.^[^ ^3^ ^]^ Increasing evidence has revealed a previously unappreciated level of complexity for human placental development. Considering the complex role of the placenta at the fetal–maternal interface, it has sparked a renewed interest to understand the dynamic roles played by placenta in regulating fetal growth. The disorders with underlying placental abnormalities not only increase maternal morbidity and mortality, but also may negatively affect fetal growth and the long‐term health of adults.^[^ ^4^ ^]^ Despite its basic and clinical significance, human early placental development is poorly understood due to the divergence between species and the limited use of primary human tissues. The models that can reflect the key features of early human placental development, especially at early gestation, are still lacking.

In the past decade, one of the most exciting advancements has been fueled by the progress of stem cells which can differentiate into any cell of an organism under certain induction microenvironments. The stem cells hold great promise for studies in disease modeling, regeneration medicine, and drug discovery.^[^ ^5^ ^]^ Especially, the advances in human pluripotent stem cells (hPSCs) have made it possible to differentiate into trophoblasts cells in 2D monolayer culture, with fine control over chemical factors,^[^ ^6^, ^7^, ^8^, ^9^, ^10^ ^]^ colony size and colony density.^[^ ^11^, ^12^ ^]^ In addition, human trophoblast stem cells (hTSCs) generated from TE and first trimester placentas are genetically stable and fulfill four characteristics of first‐trimester trophoblast.^[^ ^13^ ^]^ However, hTSCs lines are mostly grown as monolayer state without supporting matrix, which still cannot simulate the complex morphology and structure of early placental villi.^[^ ^14^ ^]^ In vivo, the extracellular matrix (ECM) can regulate the morphogenesis of maternal and placental tissues, which is crucial for supporting fetal development. The natural ECM provides scaffolds for tissue and facilitates communication between cells and their microenvironment to guide cellular adhesion, migration, and invasion at early gestation. Recently, trophoblast organoids generated from primary human trophoblast cells to model maternal–fetal interactions during human placentation have been successfully established.^[^ ^15^, ^16^ ^]^ In addition, naïve PSC‐derived trophoblast tissues and blastoids with trophoblast lineage have been well‐established.^[^ ^17^, ^18^, ^19^ ^]^ However, a better understanding of whether the differentiation of primed PSCs into trophoblasts tissue within a 3D culture condition remains elusive.

In this study, we report the formation of trophoblast‐like tissue from hPSCs in a biomimetic 3D culture condition, which can recapitulate the early placental development in vitro. hPSCs were observed to self‐organize into blastocoel‐like cavities in 3D Matrigel, which are composed of TE‐like cells. These progenitors gradually grow into millimeter‐sized 3D cultures, accompanied with the sequential differentiation into different trophoblast subtypes, including cytotrophoblasts (CTBs), syncytiotrophoblasts (STBs), and invasive extravillous trophoblasts (EVTs). Moreover, the 3D cultures exhibit characteristic structures of early placenta such as placental villi and secrete human placenta‐specific hormones. Single‐cell RNA sequencing (scRNA‐seq) analysis identified the major cell types of trophoblasts cells as existed in vivo. This study reveals the capability of establishing trophoblast‐like tissue model from the differentiated human PSCs in 3D, not accessible by 2D monolayer culture. This would provide new opportunity and model system for the study of stem cell biology, human embryology, and placental development in normal and diseases.

## 2 Results

### 2.1 Generation of Human Trophoblast‐Like Tissue from Human Pluripotent Stem Cells

**Figure 1:**
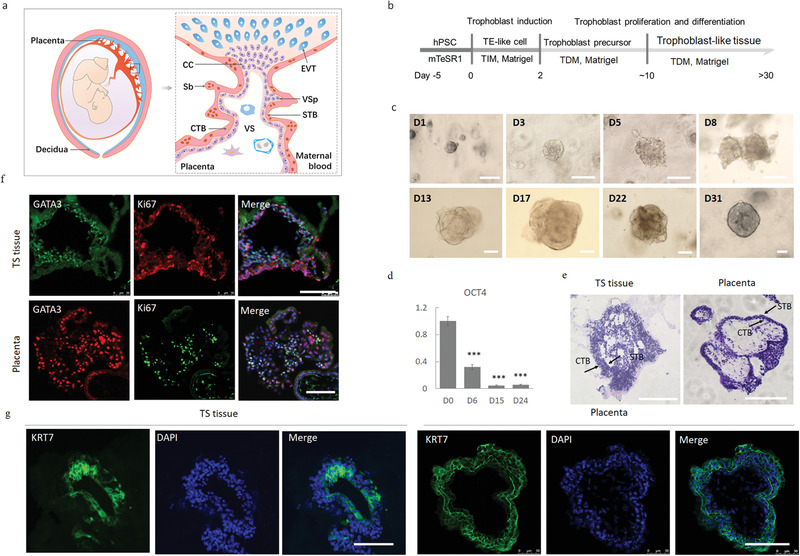
Generation of human trophoblast‐like tissue from hPSCs. a) Schematic illustration of a placental villous anchored to the maternal decidua during first‐trimester gestation. The inner CTBs undergo cell fusion to produce the outer STB layer, which is in direct contact with maternal blood. EVTs reside outside the villi and invade maternal decidua. Villous sprout (VSps) and syncytiotrophoblast buds (Sbs) are early structures for placental villi. CC: cell column; VS: villous stroma; STB: syncytiotrophoblast; CTB: cytotrophoblast. b) Schematic of the protocol used for the differentiation of hPSCs into trophoblast‐like tissue (TS tissue). TE: trophectoderm; TIM: trophoblast induction medium; TDM: trophoblast differentiation medium. c) Representative bright field images of TS tissue at different time points. The small trophoblastic cavity gradually differentiated into 3D structures, which could be maintained for more than one month. d) qPCR showing mRNA expression of pluripotency marker OCT4 (*n* = 3). Data are mean ± SD. Students t‐test, *p < 0.05, **p < 0.01, ***p < 0.001. Three independent experiments were performed. e) Representative H&E staining pictures of TS tissue at day 18 and first‐trimester placenta (Placenta) (*n* = 3; 6–8 weeks gestation), revealing densely packed cell clusters of epithelial origin with fused regions in the center of TS tissue. f,g) Immunofluorescence of tissue sections of TS tissue at day 18 and first‐trimester primary placenta (*n* = 3; 6–8 weeks gestation), showing GATA3+ cells, Ki67+ cells, KRT7+ cells, and the nuclei (DAPI, blue). Scale bars: 100 µm (c,f,g), 200 µm (e). The experiments were repeated independently three times.

In vivo, natural ECM components contain proteins such as laminin, heparan sulfate proteoglycans, and a number of growth factors. As Matrigel are rich in ECM proteins and often utilized as a 3D matrix for cell culture, we initially explored the differentiation of hPSCs under 3D in Matrigel. Specifically, hPSCs were dissociated into single cells and cultured in 3D matrix (Matrigel) (**Figure** **1**a,b). First, BMP4 was used to drive trophoblast differentiation from hPSCs in 3D condition. Notably, hPSCs self‐organized into blastocoel‐like cavities in this 3D culture condition, which were made of a dozen tall, columnar E‐cad+ cells (Figure S1a,b, Supporting Information), reflecting the intrinsic luminogenic property of hPSCs as reported in previous studies.^[^ ^20^, ^21^ ^]^ The intact cavities were confirmed by a continuous ZO‐1 signal along the cell boundary (Figure S1c, Supporting Information). Almost all cavities displayed a squamous epithelial morphology with reduced thickness during differentiation, and statistical analysis of cell nuclei demonstrated that these cavities featured flattened, laterally elongated cell nuclei (Figure S1d–f, Supporting Information). In human blastocysts, the outer layer of the conceptus consists of OCT4+/CDX2+ cells collectively called TEs. These cells give rise to proliferative CTBs after implantation and finally form a complex placental tree as in vivo (Figure S2, Supporting Information)

Interestingly, the cavitic epithelium highly expressed OCT4 and CDX2 especially at day 2, similar to TE in blastocysts (Figure S3, Supporting Information). Then, these OCT4+/CDX2+ cells gradually transitioned to CDX2+/P63+ CTB‐like cells from day 4. To test whether these precursors could proliferate and organize into complex 3D tissue, we used the defined culture conditions with chemical cocktails to explore the proliferation of trophoblast precursors in 3D (Figure 1b–d and Figures S4 and S5, Supporting Information). Surprisingly, the OCT4+/CDX2+ precursor populations at day 2, but not CDX2+/P63+ CTB‐like cells at day 4, could self‐organize into millimeter‐sized 3D tissue after 2 weeks of culture. The results suggested the important role of the cellular state at different time points in the generation of trophoblast‐like tissue. Similar to primary placental microvilli, the tissue exhibited a high proportion of GATA3+ and Ki67+ proliferative trophoblasts, and formed characteristic cavity‐like structures (Figure 1e–g). Even after freezing and thawing, high cell viability and CTB was observed in the tissue, as assessed by flow cytometry and immunofluorescence staining (Figure S6, Supporting Information), suggesting the self‐renewal capability of 3D tissue in this defined condition. Notably, the 3D tissue could be reproducibly generated from both a human embryonic stem cell (hESC) line and two human induced pluripotent stem cell (hiPSCs) lines in this optimized system (Figure S7, Supporting Information).

### 2.2 Trophoblast‐Like Tissue Expresses Markers of Cytotrophoblasts Identity, Stemness, and Proliferation

**Figure 2:**
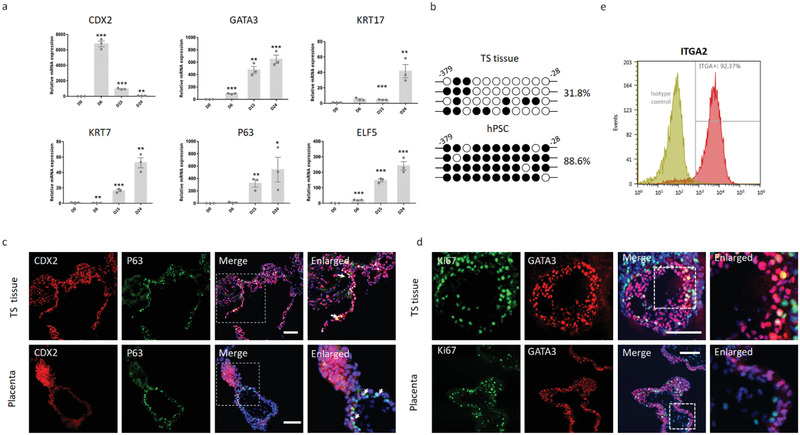
Trophoblast‐like tissue expresses markers of CTB identity, stemness, and proliferation. a) Real‐time PCR showing mRNA expression of CTB identity and stemness markers in trophoblast‐like tissue (TS tissue) at 6, 15, and 24 days of differentiation. hPSC was set as the control group (D 0). Three samples of each group were collected from two independent experiments. Data are mean ± SEM. Students t‐test, *p < 0.05, **p < 0.01, ***p < 0.001. b) Bisulfite sequencing of the ELF5 promoter region of TS tissue and hPSCs (positive control). The relative percentage of methylated cytosine residues (filled circles) is indicated. c,d) Representative pictures of TS tissue (day 31) and first‐trimester placenta (Placenta) (*n* = 3; 6–8 weeks gestation) showing CDX2, P63, Ki67, GATA3, and nuclei (DAPI, blue). The square area was enlarged as shown on the right. White arrows indicate CDX2+ and P63+ cells. e) FACS analysis of TS tissue stained with anti‐ITGA2 (Red), representing the cells at the base of the cytotrophoblast cell columns. The experiments were repeated independently three times. Scale bars: 100 µm.

To further assess the identity of the formed trophoblast‐like tissue in the 3D culture condition, we examined widely accepted criteria for human trophoblasts including DNA methylation, cell surface antigens (HLA class I), and transcription factors (TFs) of human CTB identity and stemness^[^ ^22^ ^]^ (**Figure** **2**a,b and Figures S5 and S8, Supporting Information). All CTB markers displayed typical expression patterns with a few exceptions (e.g., FZD5 and TEAD4) when compared to hPSCs. However, the FZD5 and TEAD4 genes were also downregulated in the primary placenta (Figure S5, Supporting Information). After 6 days of differentiation, the OCT4 signal was undetectable accompanied by increased expression of P63 (Figure 2c,d and Figure S9, Supporting Information). Therefore, the OCT4+/CDX2+ trophoblast precursor^[^ ^23^ ^]^ gradually transitioned to CDX2+/P63+ CTBs from day 6, which was in agreement with trophoblast differentiation during placental development in vivo. Different from physiological features, P63+/Ki67+ CTBs lined along the cavity with an inside–out architecture. Specifically, these cells were on the outside of the cavity and in direct contact with Matrigel. To confirm a proliferative trophoblast niche,^[^ ^24^ ^]^ flow cytometry was performed which revealed >90% ITGA2+ (CD49b) cells in the 3D tissue (Figure 2e). The percentage of ITGA2+ cells gradually decreased to ≈60% during the process of cell differentiation, accompanied by the downregulation of proliferative cell marker Ki67 (Figure S10, Supporting Information). A high proportion of proliferating trophoblast precursors implied the high potential of these self‐organizing 3D tissue to generate both villous and EVT lineages.

### 2.3 Trophoblast‐Like Tissue Contains Trophoblast Subtypes Syncytiotrophoblasts and Extravillous Trophoblasts

**Figure 3:**
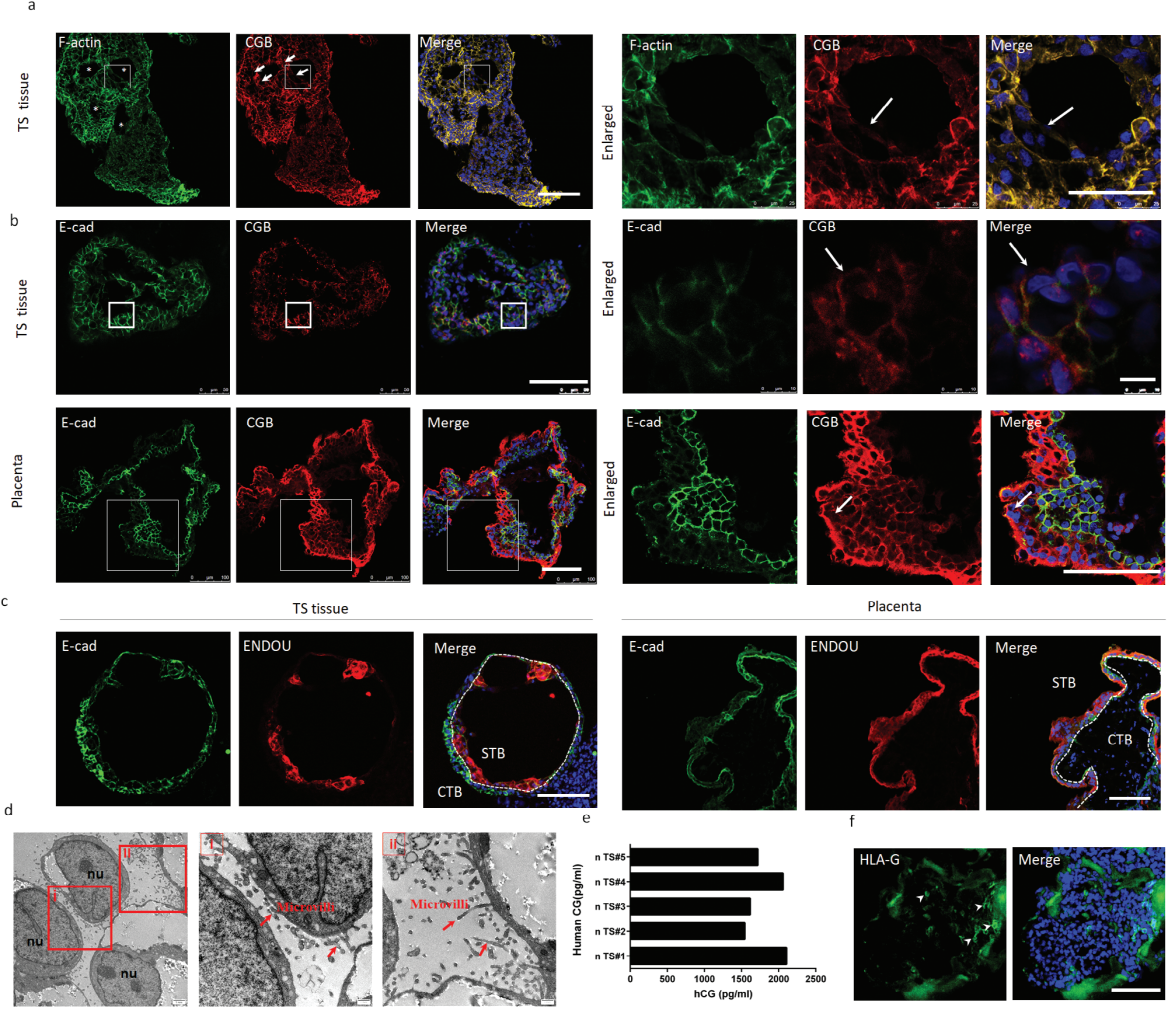
Trophoblast‐like tissue contains trophoblast subtypes STB and EVT. a) Fluorescence image of cavity‐like structures (white asterisks) in 3D tissue at days 33 which are characterized by the expression of F‐actin and CGB (an STB marker). The white square highlights multinucleated STB (white arrowheads) shown in the enlarged image. Multinucleated STB indicated the cell fusion of TS tissue. b,c) Representative pictures of trophoblast‐like tissue (TS tissue) (day 18 [b]; day 33 [c]) and first‐trimester placenta (Placenta) (*n* = 3; 6–8 weeks gestation) showing CGB, E‐cad, and ENDOU. White arrowheads indicate multinucleated STB and white arrows. d) Representative electron transmission microscopy images of the TS tissue. The square areas were enlarged as shown on the right. Red arrowheads indicate microvilli on the surface of the TS tissue. nu: nucleus. e) ELISA for hCG‐*β* secreted by TS tissue. The amount of hCG‐*β* (pg mL^−1^) produced by TS tissue (*n* = 5 time points during the differentiation of 3D tissue from day 10 to day 25) at 48 h is shown. Supernatants from TS tissue cultures at different time points were derived independently from two experiments. f) Representative pictures of TS tissue showing the EVT marker HLA‐G at days 33. White arrow heads indicate sporadic HLA‐G+ cells. Scale bars: 100 µm. Scale bars: 50 µm (a‐[Enlarged]) and 10 µm (b‐[Enlarged]) in TS tissue.

Next, trophoblast subtype STBs and EVTs were investigated to examine the cellular components within the 3D tissue. Clearly, the upregulation of STB‐ and EVT‐specific markers was detected using quantitative PCR (qPCR) (Figures S11 and S12, Supporting Information). Similar to primary placental tissue at 6 to 8 weeks of gestation, the generated 3D trophoblast‐like tissue (TS tissue) in vitro contained cavity‐like structures formed by multinucleated STBs (**Figure** **3**a–c and Figure S11b, Supporting Information), implying the crucial role of a 3D culture in promoting STB differentiation.^[^ ^13^, ^25^ ^]^ In contrast, the 3D tissue displayed an inverse organization with multinuclear STBs lining the central cavity, suggesting that CTBs undergo cell–cell fusion toward the inner part. Consistent with the inside–out architecture of trophoblast‐like tissue, ENDOU were expressed in the outer STB layer of the primary placental villi and in the center of the cavity (Figure 3c). Moreover, the characteristic feature of placenta in TS tissue was investigated by transmission electron microscopy, in which cells with surface microvilli were detected (Figure 3d). hCG*β* is a clinical marker for pregnancy, and is often secreted into the maternal circulation by STB in the human placenta. Herein, hCG*β* in the culture supernatant was measured by ELISA (Figure 3e), indicating the function of the generated tissue in secreting hCG*β* in 3D cultures. Generally, invasive trophoblast‐derived EVT is crucial for pregnancy success as it invades the maternal decidua to transform the spiral arteries.^[^ ^26^ ^]^ Our 3D system also showed obvious EVT differentiation defined by the molecular marker HLA‐G (Figure 3f and Figure S12d, Supporting Information), verifying the similar cellular components in the trophoblast‐like tissue to those in primary placental tissue.

### 2.4 Bioinformatic Analysis of Trophoblast‐Like Tissue and Primary Placental Villi

**Figure 4:**
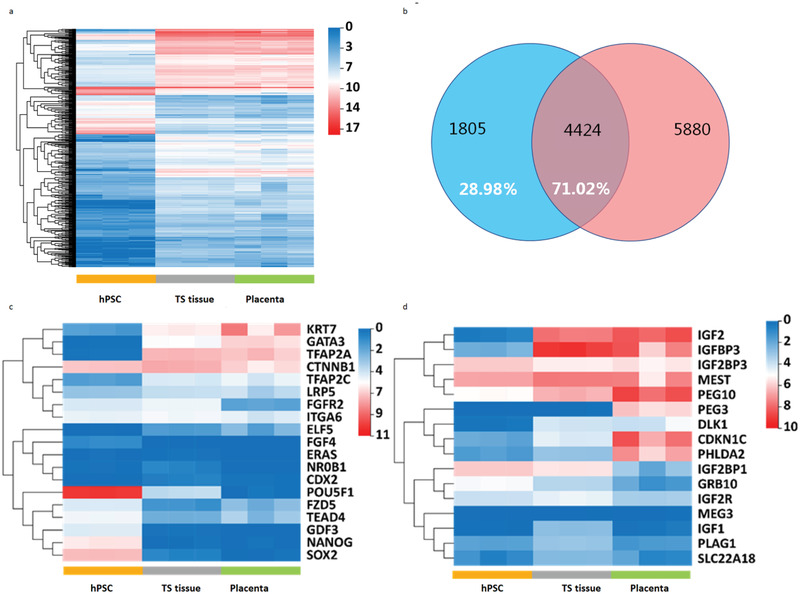
Trophoblast‐like tissue retains similar transcriptomic profiles with first trimester trophoblast (6–8 weeks gestation). a) Clustered heat map of differentially expressed genes in first trimester placental villi, trophoblast‐like tissue (TS tissue) at day 24, and cultured hiPSCs. The primary placenta (Placenta) (6–8 weeks, *n* = 3) used in this study for analysis were collected from three patients. TS tissues (*n* = 3) were from three independent experiments. Analysis is based on *p* < 0.001. b) Venn diagram shows that of 6229 differentially expressed genes (DEGs) in TS tissue (greater than twofold change relative to hPSC; *p* < 0.001), 4424 were common to DEGs between hPSCs and placental villi. c) Clustered heatmap depicting selected expression patterns of markers of pluripotency and CTB stemness. CTB stemness genes TFAP2A and TFAP2C show high expression levels across the TS tissue and placenta, while very low or no expression in hPSCs. By contrast, pluripotent genes including POU5F1 (OCT4), NANOG, and SOX2 displayed very low or no expression across the TS tissue and placenta, while very high expression in hPSCs. These expression patterns suggested the efficient differentiation toward trophoblast in TS tissue. d) Heat map of key imprinted genes with significant functions in embryonic development. TS tissue exhibited very similar gene expression patterns to first‐trimester placenta. The gene expression refers to fragments per kilobase million (FPKM).

To gain a global view of the gene expression profiles of the 3D culture, transcriptome analysis was performed between undifferentiated hPSC, trophoblast‐like tissue (TS tissue), and primary 6–8 weeks placenta tissue (Placenta). We selected ≈700 genes with higher expression in 3D tissue than in hPSCs and subjected them to hierarchical clustering (**Figure** **4**a and Table S2, Supporting Information). The results revealed a unique gene set enriched in 3D tissue and first trimester placental villi in comparison to the hPSCs. Impressively, 71.02% of trophoblast‐like tissue upregulated genes (greater than twofold change relative to hPSCs, adjusted *p* ≤ 0.001) were common to primary placenta (Figure 4b,c, and Figure S13a,b, Supporting Information), including a series of markers for CTB identity and stemness, EVT and STB. Compared with trophoblast differentiation, the expression of pluripotent genes was significantly downregulated in agreement with the above qPCR and immunofluorescence data. Upon comparison of the primary placenta, EVT and STB markers showed a moderate expression level in the TS tissue, possibly revealing the fact that the stage represented by TS tissue developed earlier than the examined placental villi (6–8 weeks of gestation).

Transcription factor (TF) genes from the TF ELF family including ELF5 were upregulated, validating the hypomethylation of the gene promoter (Figure S13c, Supporting Information). Additionally, differentially expressed genes (DEGs) in the 3D tissue highlights other genes of interest in addition to the known TF genes GATA3, P63 (TP63), and TFAP2A (Figure S13d, Supporting Information). The comparison of DEGs (fold change ≥ 2, adjusted *p* ≤ 0.05) among hPSCs, TS tissue, and placenta highlighted other genes of interest, such as, HAND2, SNAI2, TWIST1, PITX1, and ID2. In particular, almost all key imprinted genes exhibited similar expression patterns in the 3D tissue and placental tissue including IGF2, MEST, PEG3, PEG10, DLK1, and PHLDA2 (Figure 4d and Table S3, Supporting Information). These genes have been reported to have a strong correlation with human placental functions and fetal growth.^[^ ^27^, ^28^ ^]^ Moreover, consistent with the gene expression pattern, the IGF2 protein was also secreted from the 3D tissue (data not shown).

### 2.5 Single‐Cell Transcriptomics Analysis of Trophoblast‐Like Tissue Differentiation

**Figure 5:**
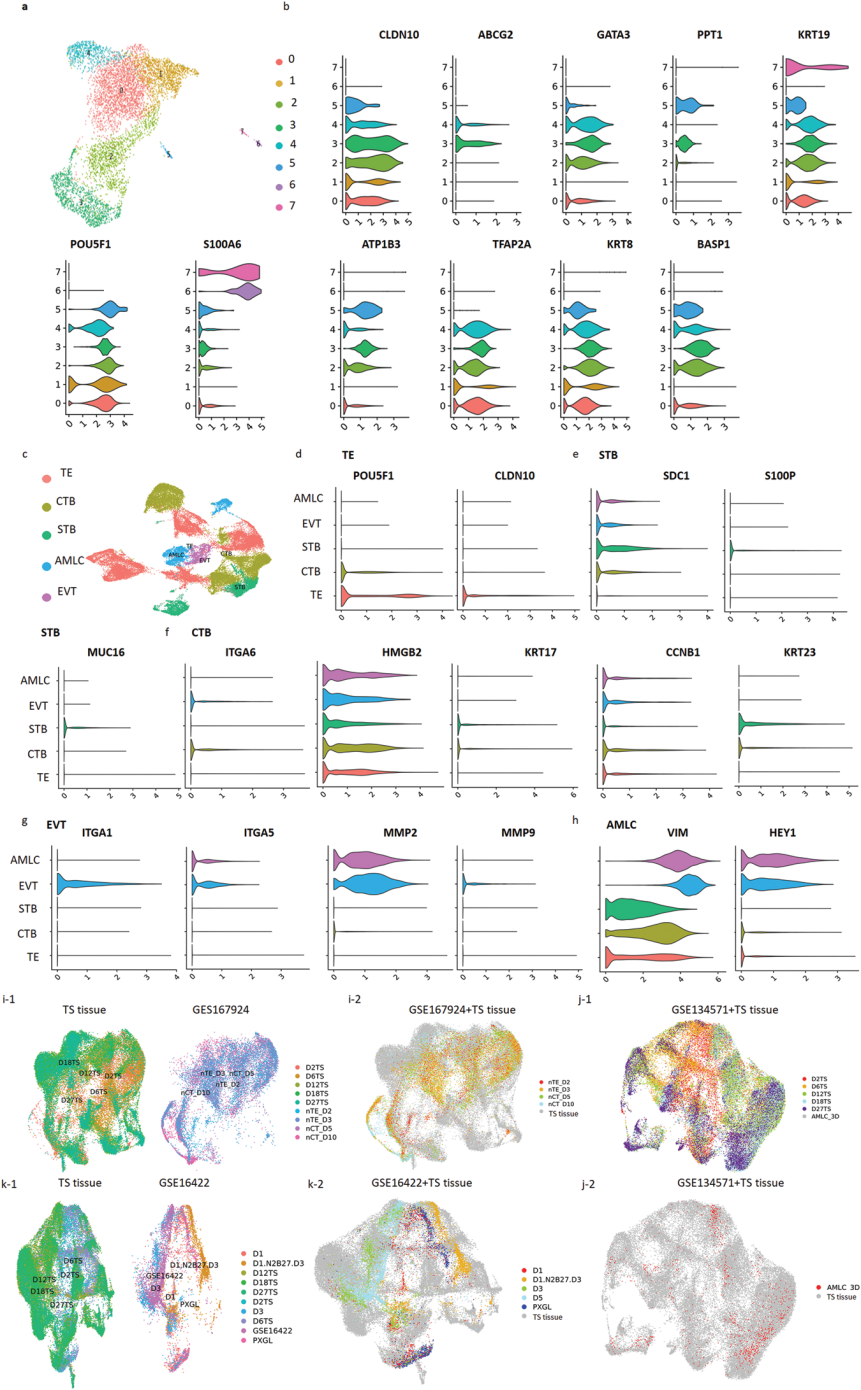
Single‐cell transcriptomics analysis of trophoblast‐like tissue differentiation. a) UAMP plot displaying 6753 cells from the 10× Genomics scRNA‐seq analysis after 2 days of differentiation. Unsupervised clustering identified three clusters that are marked by different colors. The seven clusters were subsequently classified into TE according to cell‐specific markers. TE. b) Violin plots showing the expression of representative TE‐specific marker genes within the trophoblast subclusters after 2 days of differentiation. c) UAMP plot displaying 44825 cells from the 10× Genomics scRNA‐seq analysis at 2, 6, 12, 18, and 27 days of differentiation. Unsupervised clustering identified 15 clusters that are indicated by different colors. The 15 clusters were subsequently classified into five subclusters according to cell‐specific markers. d–h) Violin plots showing the expression pattern of specific genes among different cell types of trophoblast tissue including TE (d), STB (e), CTB (f), EVT (g), and AMLC (h). TE was characterized by the expression of CLDN10 and POU5F1. STB was characterized by the expression of SDC1, MUC16, and S100P. CTB was characterized by the expression of ITGA6, KRT17, KRT23, HBMG2, and CCNB1. EVT was featured by the expression of ITGA1, ITGA5, MMP9, and MMP2. AMLC were featured by the expression of VIM1 and HEY1. TE: trophectoderm; CTB: cytotrophoblast; STB: syncytiotrophoblast; EVT: extravillous trophoblast; AMLC: amnion‐like cells. i‐1,i‐2) Comparison of the TS tissue's ScRNA‐seq data with naïve hPSC‐derived trophoblast (GSE167924). The UAMP plot of TS tissue and naïve hPSC‐derived trophoblast, respectively (i‐1). The TS tissue clustered with naïve hPSC‐derived trophoblast (i‐2). j‐1,j‐2) Comparison of the TS tissue's ScRNA‐seq data with hPSC‐derived amnion (GSE134571). The merge of TS tissue and hPSC‐derived amnion cells (j‐1). The TS tissue clustered with AMLC in the hPCS‐deived amnion tissue (j‐2). k‐1,k‐2) Comparison of the formed TS tissue's ScRNA‐seq data with naïve hPSC‐derived trophoblast (GSE16422). The UAMP of TS tissue and naïve hPSC‐derived trophoblast, respectively (k‐1). The TS tissue clustered with naïve hPSC‐derived trophoblast (k‐2).

To gain more insight into the trophoblast‐like tissue (TS tissue), scRNA‐seq on different days of differentiation were conducted (Figures S14–S17, Supporting Information). Using the 10× Genomics Chromium, mRNA from 7076, 11163, 9152, 10626, and 9968 cells harvested on days 2, 6, 12, 18, and 27 of differentiation were sequenced, respectively. As shown in Figure S3, Supporting Information, the cavitic epithelium was OCT4+/CDX2+ TE‐like cells. Then, we further analyzed cells from 2 days of differentiation to better understand the cellular identity in the cavity. According to previously published datasets of TE lineage‐specific marker, the TE‐related markers refer to POU5F1, CLDN10, S100A6, GATA2, ATP1B3, SOX4, TBX3, FGFR4, etc. in this study.^[^ ^29^, ^30^ ^]^ We therefore annotated and refined that the majority of cells are TE‐like cells (Figure **5**a), which were characterized by the high expression of marker genes specific to TE (Figure 5b). Additionally, we detected no expression of polar TE‐specific genes, such as CCR7, CYP19A1, MUC15, and OVOL1 in the D2 tissue (Figure S18, Supporting Information).

Cell ranger (version 3.1.0) was used to perform clustering based on the gene expression level. Unsupervised clustering of the entire pooled dataset of 44825 cells from D2, D6, D12, D18, and D27 indicated a total of 15 transcriptionally distinct clusters, as visualized in a uniform manifold approximation and projection (UMAP) plot (Figure 5c). According to previous studies, the specific markers of CTB refer to ITGA2, ITGA6, ITGB4, CCNB1, KRT23, KRT17,^[^ ^14^, ^18^ ^]^ etc. Additionally, the CTB is characterized by the expression of proliferation‐related genes MKI67, TOP2A, CCNB1, AURKB, ASPM, CENPF, and HMGB2.^[^ ^31^ ^]^ The STB‐related markers refer to CGA, SDC1, S100P, MUC16, and CD63.^[^ ^14^, ^19^, ^32^ ^]^ The EVT‐related markers refer to HLA‐G, CD9, MMP2, MMP9, ITGA1, and ITGA5.^[^ ^14^, ^19^ ^]^ The 15 clusters were clustered into six broad classes of cells including TE, CTB, STB, EVT, and others clusters, by comparing specific transcription profiles. The other clusters are characterized by the expression VIM, GABRP1, IFGP5, ISL‐1, and HEY1 which has been reported in amnion cells.^[^ ^18^ ^]^ We thus defined these clusters amnion‐like cells (AMLC). Violin plots demonstrate the expression of representative marker genes across different clusters (Figure 5d–h). The biaxial scatter plots show the expression of representative marker genes across different clusters of TS tissue from D6, D12, D18, to D27 (Figures S14–S17, Supporting Information).

Nowadays, the differentiation of amnion lineage and trophoblast lineage were still controversial. To investigate the relationship of the generated 3D tissue with other trophoblast tissues and amnion cells, we conducted a comparative analysis with published single‐cell transcriptomes derived from naïve hPSC‐derived trophoblast cells (GSE167924 and GSE16422), naïve hPSC‐derived TE (GSE167924), and primed hPSC‐derived amnion cells (GSE134571). The trophoblast‐like tissue was clustered with majority of hPSC‐derived TE (Figure 5i) and trophoblast cells (Figure 5i,k), and minority of hPSC‐derived amnion cells (Figure 5j).

Additionally, to reconstruct lineage relationships, Monocle 2 was used to preform unsupervised lineage trajectory analysis. The pseudotimes were well matched with differentiation time points of TS tissue (Figure S19a–c, Supporting Information). We also probed DEGs corresponding with the bifurcation at the first branchpoint. The top 50 DEGs demonstrated that this branchpoint was enriched with genes involved in trophoblast stem cell differentiation and placental development, such as CDH1, TEAD3, and FUT8 (Figure S19d, Supporting Information). These data reflect that trophoblast lineages within TS tissue emerge from a common origin at the onset of placentogenesis. These results further verified that TS tissue differentiation in vitro follows an orderly sequence of processes involving the emergence of TE, CTB, STB, and EVT, which is consistent with observations during primary placental development.

## 3 Discussions

In this work, we describe the establishment of trophoblast‐like tissue (TS tissue) model in vitro from the differentiation of hPSCs in a 3D culture system. This tissue model possesses the major cell types of trophoblasts including CTB, EVT, and STB. In particular, it could resemble several key features of villous placental development at an early stage, including cellular subtypes, microvilli structures, and hormone secretory ability, which have not been observed in cell monolayer culture.^[^ ^6^ ^]^

Current studies have reported the ability of hPSCs differentiation into trophoblast in 2D monolayer cell culture.^[^ ^6^ ^]^ hTSCs lines are genetically stable and fulfill all four characteristics of first trimester trophoblast. However, the cells grown in 2D culture could not faithfully recreate the complex structure of early placental villi. In this study, we demonstrated the possibility of primed hPSCs differentiation into the in vivo‐like trophoblast tissues in 3D matrix, suggesting the crucial role of 3D ECM to mimic the near‐physiological microenvironment and guide the development of TS issue from hPSCs.

Notably, blastocoel‐like cavities could be efficiently generated, which were identified to be composed of TE‐like cells. As observed in vivo, TE‐like cells were found to differentiate into trophoblast progenitors CTB, which finally matured into STB and EVT. In line with the recently published articles which reported the presence of trophoblast lineage in the generated blastocyst‐like structure,^[^ ^19^, ^33^ ^]^ these data reflected the spatial and temporal differentiation of hPSC into trophoblast subtypes in the presented system. However, the established early blastocoel‐like cavities exhibited an inside–out architecture that was inverted to in vivo blastocyst TE. Previous studies have reported the intrinsic property of hPSCs to form apico‐basally polarized cavities,^[^ ^20^, ^21^ ^]^ but they often showed an inverted organization compared to that in vivo. As thus, we speculate the early TS tissue may not fully recreate in vivo blastocyst‐like morphology using hPSCs due to the intrinsic self‐organization property of stem cells without better control of microenvironment.

Previous studies indicated that that naive hPSCs can give rise to trophoblast lineage^[^ ^19^, ^33^ ^]^ and recapitulate trophoblast development^[^ ^17^, ^18^ ^]^ under defined culture condition, while the BMP‐treated primed hPSCs resemble amnion cells.^[^ ^34^ ^]^ To further characterize the lineage development, we compared previously published scRNA‐seq dada of human naïve PSC‐derived TE,^[^ ^18^ ^]^ human naïve PSC‐derived trophoblast,^[^ ^17^, ^18^ ^]^ and hPSC‐derived amnion.^[^ ^34^ ^]^ It revealed that the 3D tissue shared common genes of trophoblast cells and amino cells, revealing that the generated tissue might be a mixture of trophoblast and amnion cells. However, the identification of amnion cells was still questionable due to the lack of well‐established amnion‐specific marker. Further work is needed to further exemplify the existence of amnion cells. In addition, we detected low secretion of the h‐CG, which is not as high as the level of human chorionic gonadotropin (hCG) concentration in vivo during pregnancy. We assume that this might be resulted from the immature state of STB in the tissue.

Decades of studies into placental development have revealed the high complexity with respect to the dynamic microenvironment during pregnancy, which includes biophysical and chemical cues and integration of these signals to become a functional organ.^[^ ^35^ ^]^ Increasing evidence has demonstrated the vital role of these mechanical cues in the differentiation and maturation of human organs and tissues.^[^ ^36^, ^37^ ^]^ A human amnio tissue were generated from the self‐organization of hiPSCs in a perfused 3D culture system ^[^ ^37^ ^]^ This chip system provided biometric mechanical cues such asdynamic flow for the differentiation of hiPSCs toward amino tissue under 3D matrix. The generated amnion recapitulates the development of amino in midgestation, thus revealing the importance of microenvironment. Therefore, further work can be done to explore trophoblast differentiation from hPSCs in vitro under various mechanical cues, such as ECM rigidity and dynamic flow combination with signaling molecules such as BMP4 which are essential for the differentiation of trophoblast cells. Particularly, mechanical cues at the human maternal–fetal interface, such as tissue stiffness, could greatly affect placental development, thereby potentially resulting in normal or diseased pregnancy. Thus, our deep understanding of trophoblast tissue model from hPSCs might contribute to the study of developmental biology and gestational disorders.

## 4 Conclusions

In summary, we described the differentiation capability of primed hPSCs to extraembryonic trophoblast‐like cells, placental microvilli, and even trophoblast‐like tissue (TS tissue) in 3D cultures. The generated TS tissue in 3D has unique advantages in terms of unveiling the developmental processes of the human early placenta, such as from TE to a subset of trophoblast subtypes, which is not accessible by other monolayer culture. It may provide a new in vitro model system to study the physiological interactions at the maternal–fetal interface, as well as the metabolic and endocrine activities in human placentation. We envision this new model system is helpful to investigate the early placental maldevelopment associated with pregnancy diseases, such as preeclampsia, fetal death, and fetal growth restriction, which may potentially contribute to the study of placentogenesis, human embryology, and gestational disorders.

## 5 Experimental Section

### Tissue Collection

This research was conducted under ethical approval from the Ethics Committee on ART of Dalian Municipal Women and Children's Medical Center (2019005). Based on the guidelines in the Declaration of Helsinki by the World Medical Association, written informed consent from all participants was obtained. All placental tissues (6–8 weeks gestation) used for this study were obtained from elective terminations of normal pregnancies (vacuum aspiration) without medical indication in Dalian Municipal Woman and Children's Medical Center. After washing with 0.9% NaCl, the primary placenta was stored in PBS buffer for immediate use.

### Cell Lines and Culture

hESC (H7) and two hiPSC lines (produced from skin fibroblasts) were utilized in this study, which were kind gifts from Dr. Ning Sun (Fudan University, China). hPSC lines were cultured under feeder‐free conditions and maintained in mTeSR1 medium on Matrigel‐coated plates. They were passaged at a 1:5 ratio every 4–5 days using Accutase. More specific details were described in the authors' previously published studies.^[^ ^38^ ^]^

### Generation of Trophoblast‐Like Tissue from Human Pluripotent Stem Cells

To generate trophoblast‐like tissue (TS tissue), hPSCs were digested into single with Accutase and then resuspended with Matrigel (BD Bioscience) at a concentration of ≈1 × 10^6^ cells/ml (Day 0). Cell drops (≈20 µL) were plated in 12‐well plates and maintained at 37 °C for about 30 min. After gelation, cells were cultured in trophoblast induction medium containing DMEM/F12, 2% BSA, 1× ITS (Sigma), 1× MEM‐NEAA, 1× L‐GlutaMAX, 100 ng mL^−1^ heparin, 1× penicillin–streptomycin with 10 ng mL^−1^ BMP4 for 2 days. At day 2, cells were cultured in trophoblast tissue medium (TDM) containing DMEM/F12, 1× N2, 1× B27 minus Vitamin A, 1.25 mm *N*‐acetyl‐L‐cysteine, 1× L‐GlutaMAX, 1× penicillin–streptomycin with 500 nm A83‐01, 1.5 µm CHIR99021, 50 ng mL^−1^ human EGF, 80 ng mL^−1^ human R‐spondin 1, 100 ng mL^−1^ human FGF2, 50 ng mL^−1^ human HGF, 2 µm Y‐27632, and 2.5 µm PGE2 (Table S4, Supporting Information). Medium was changed every 2–3 days. Small 3D clusters were passaged at a ratio of 1:5 when 3D tissue reached a diameter of ≈200 µm (usually at day 10). Tissue‐containing domes were mechanically disrupted by pipetting up and down for dozens of times (≈200 times). Following cell harvest by centrifuging, cells were resuspended with Matrigel. To examine trophoblast differentiation, cells were treated with BMP4 for 1, 2, 3, and 4 days, respectively, and then cultured with TDM. For cryopreservation, freezing solution containing 70% TDM, 20% FBS, and 10% DMSO were prepared to resuspend cell clusters, and finally stored in liquid nitrogen after frozen at −80 °C for 1 day. To thaw TS tissue, cell stocks were thawed quickly at 37 °C in water bath, and resuspended in TDM following cell harvest by centrifuging.

### Tissue Preparation and Immunofluorescence Staining

More specific details were described in the authors' previous studies.^[^ ^38^ ^]^ Specifically, TS tissue at desired days of differentiation were fixed in 4% paraformaldehyde for 20 min at room temperature. After washing with PBS for three times, tissues were dehydrated by incubation with 30% sucrose overnight at 4 °C for dehydration. Then, the tissues were embedded in O.C.T. (SAKURA) and cryosectioned at 15–20 µm using a cryostat (Leica). Samples on adhesive slides were washed by immersion in PBS before the immunofluorescence staining. Frozen sections were permeabilized with 0.2% Triton X‐100 before blocking with goat serum (ZLI‐9022, ZSGB‐Bio) for 1 h at room temperature. After washing with PBST (PBS with 0.05% Triton X‐100), samples were incubated with primary antibodies overnight at 4 °C, and secondary antibodies for 1 h at room temperature. For more details in antibodies used, see Table S5, Supporting Information. After counterstaining of nuclei with DAPI, samples were imaged using a confocal microscope (Olympus). For samples between days 1 and 4, tissues were directly incubated with antibodies after cell permeabilization and prepared to image. For staining F‐actin, samples were stained with phalloidin, CF‐488A (00042, Biotium) according to the manufacture's instruction.

### ELISA

To examine hCG*β* secreted from TS tissue, hCG ELISA (Abcam, ab100533) was utilized. Cell medium was collected in centrifuge tube (Jet Biofil) after culture for 48 h and centrifuged to remove debris. Medium was stored at −80 °C until use. Samples were prepared following the instructions provided by the manufacturer and measured by TECAN Infinite M Nano.

### Flow Cytometry

3D tissue was extracted from the gel with Cell Recovery Solution (Corning 354253). Following incubation at 4 °C for 20 min to depolymerize Matrigel, cell spheroids were dissociated with 0.25% trypsin at 37 °C for 10 min. Single cells were obtained by filtering through 40 µm cell strainers (Falcon 2340) after washing with medium containing FBS. Finally, cells were labeled with HLA‐G–PE, ITGA2–PE, or isotype‐matched controls for analysis (Table S5, Supporting Information). Cell viability was analyzed using LIVE/DEAD Fixable Near Red Dead Cell Stain (Life Technologies L10119). The experiments were conducted using SH800S Cell Sorter (Sony Biotechnology). Data were analyzed in Cell Sorter Software (Sony Biotechnology).

### Transmission Electron Microscopy

TS tissue samples were collected and then fixed with polyformaldehyde and glutaraldehyde (2.5% in PBS; pH = 7.4) at room temperature for 1 h. After that, tissues were postfixed for 1.5 h with 1% osmium tetroxide insodium cacodylate buffer (0.1 mol L^−1^), and stained with 1% w/v uranyl acetate for 50 min in double distilled water. After washing and dehydration, samples were embedded in Embed 812 resin and polymerized for 48 h at 60 °C. Polymerized samples were trimmed and sliced to 50 nm sections using a Leica ultramicrotome EM UC6 (Leica, Germany). EM grids coated with carbon film were used to collect on the sections. Spirit transmission electron microscope (FEI Company, the Netherlands) (operating at 100 kV) were used to image for TEM sample grids.

### DNA Extraction and Quantification

Genomic DNA from hPSC and TS tissue samples for bisulfite sequencing were extract using the Thermo Scientific GeneJET Genomic DNA Purification Kit (K0721, K0722). DNA was purified at the instruction of manufacture. DNA quality and concentration were finally determined in a NanoPhotometer (IMPLEN).

### Bisulfite Sequencing

250 µg genomic DNA was prepared for a bisulfite conversion using the EZ DNA Methylation‐Gold Kit (D5005, Zymo Research) at the instruction of manufacturer. The ELF5 promoter region was amplified according to the previous study.^[^ ^16^ ^]^ PCR products were cloned and 3730XL DNA Analyzer (Applied Biosystems) were used to sequence, confirming the methylation status of each CpG site.

### RNA Extraction, Reverse Transcription, and Quantitative Real‐Time PCR

More specific details were described in the authors' previous studies.^[^ ^38^ ^]^ Specifically, Trizol reagent (TAKARA) were used to isolate the total mRNA from TS tissue samples or hPSCs. RNA concentration and quality were determined by NanoPhotometer (IMPLEN), and the final concentration of RNA was adjusted to 500 ng mL^−1^. cDNA was produced from 1 mg RNA and then amplified using Ex Taq DNA polymerase (Takara) under the following reaction conditions: denaturation at 94 °C for 1 min, annealing at 58 °C for 45 s, and extension at 72 °C for 30 s; the cycle number was 35. The primer pairs used in this work are as listed in Table S1, Supporting Information. Real‐time PCR was performed on PikoReal 96 (Thermo Scientific).

### RNA‐Sequencing and Data Processing

hiPSC, TS tissue cultures, and first‐trimester placental villi (6–8 weeks gestation) were harvested to conduct gene expression analysis. Total mRNA was isolated and dissolved in RNase‐free water. RNA‐sequencing was performed using BGISEQ‐500RS (BGI Company). Bowtie2 was used to map the reads to human reference genome (Build hg19) and Edge was used to determine the differential RNA expression. Normalized levels of gene expression and the color scale were determined by scaling raw reads for each gene to account for different total read counts across samples before values were log2 transformed.

### Characterization of Cell and Cavity Morphology

Cell nucleus dimensions and cavity thickness was measured manually using Image Pro Plus software. The normalized nucleus dimension (visualized by nuclear staining with DAPI), defined as the ratio of nucleus dimension along the apico‐basal direction to that along the lateral direction, was then calculated. Cell cavity dimensions (inner and outer diameter) were measured at four different positions evenly distributed along the circumference of the cavity in bright field images. The cavity epithelial thickness equaled the data, that is, the number of outer diameters minus the number of inner diameters.

### Single‐Cell RNA‐Sequencing Library Preparation

Day 2, Day 6, Day 12, Day 18, and Day 27 TS tissue was extracted from Matrigel by incubation with ice‐cold Cell Recovery Solution (Corning 354253) for 20 min at 4 °C. Then, these 3D tissues were dissociated by digestion with 0.25% trypsine for 10 min at 37 °C. Single cells were obtained by filtering through 40‐µm cell strainers (Falcon 2340) after washing with medium containing FBS. Trypan blue were subsequently used to detect the dissociated cells and the viability of cells were at least 80% viability. According to the manufacturer's recommendations, there should be at least 6000 dissociated cells which were loaded per well for single‐cell RNA‐seq library preparation with the 10× Genomics (V3). Illumina NovaSeq (capital biotech) were employed to sequence the generated libraries and mRNA from 7076, 11163, 9152, 10626, and 9968 cells harvested on days 2, 6, 12, 18, and 27 of differentiation were sequenced, respectively.

### Single‐Cell RNA‐Seq Data Processing

According to previous studies,^[^ ^39^ ^]^ cell ranger (version 3.1.0) was employed to align sequenced reads to GRCh38 and aggregate output across all samples. The UMI counting was normalized, and PCs were selected as input for UMAP for Day 2, Day 6, Day 12, Day 18 and Day 27 cells. The Loupe Browser was used to visualize and analyze the results. 6753 Day 2 single cells, 10225 Day 6 single cells, 8116 Day 12 single cells, 9850 Day 18 single cells, and 9381 Day 27 single cells remained. Monocle2 (version 2.10.0) was used to perform unsupervised lineage trajectory analysis on a downsampled set of Day 2, Day 6, Day 12, Day 18, and Day 27 cells (6251 cells, 10% of all cells). Briefly, the authors used genes with mean expression value ≥0.05 to order cells along the trajectory. Then, branched expression analysis modeling function of Monocle2 was used to identify significant DEGs (*q*‐value % 1e‐4) at branch points along the trajectory. Significant branch specific DEGs were visualized in a branched heatmap to observe changes in both cell fates concurrently.

### Statistics and Reproducibility

All experiments indicated in this study were repeated with consistent results derived from independent samples from several patients or TS tissue. The number of samples and the time points for sample preparation are described in the figure legends. The number of times repeated for experiments are also reported in the figure legends. Data were mean ± SD or ± SEM. Student's *t*‐test. *p* < 0.05 was accepted as statistically significant. Trophoblast‐like tissue culture protocols were independently replicated by three scientists in the lab. The Excel, Origin, and GraphPad were used to process the data in this study.

## Conflict of Interest

The authors declare no conflict of interest.

## Author Contributions

K.C. and Y.Z. contributed equally to this work. J.Q. conceived, designed the study, and wrote the manuscript. K.C. and Y.Z. conducted the experiments, analyzed data, and wrote the paper. K.C. and T.C. contributed to analyzing data of RNA‐sequencing. H.W. conducted the experiments with hPSCs. Y.G. and P.D. helped to draw the pictures. H.L. helped check data. X.S. and Y.S. provided patient samples and discussed the results.

## Supporting information

Supporting Information

Supplemental Table 1

Supplemental Table 2

Supplemental Table 3

Supplemental Table 4

Supplemental Table 5

## Data Availability

Data files of RNA‐sequencing and single cell RNA‐sequencing that support the findings of this study have been deposited in NCBI Sequence Read Archive (SRA) repository (http://www.ncbi.nlm.nih.gov/sra/) with accession number PRJNA554772 and PRJNA657730. All other data that support the findings of this study are available from the corresponding authors upon reasonable request.
