# Supplementary material for: Establishment of Trophoblast‐Like Tissue Model from Human Pluripotent Stem Cells in Three‐Dimensional Culture System
Source: Adv Sci (Weinh). 2021 Nov 23;9(3):2100031. doi: 10.1002/advs.202100031 (PMC8787386; doi:10.1002/advs.202100031)
Supplement: Supplementary file 1 — Supporting Information [file ADVS-9-2100031-s002.pdf]

ADVANCED SCIENCE
Open Access

Supporting Information

for *Adv. Sci.*, DOI: 10.1002/advs.202100031

Establishment of Trophoblast-Like Tissue Model from Human Pluripotent Stem Cells in Three-Dimensional Culture System

Kangli Cui[1,4 †], Yujuan Zhu[1,4 †], Yang Shi[5], Tingwei Chen[6], Hui Wang[1,4], Yaqiong Guo[1,4], Pengwei Deng[1,4], Haitao Liu[1,4], Xiaoguang Shao[5], and Jianhua Qin[1,2,3,4]*

Establishment of Trophoblast-Like Tissue Model from Human Pluripotent Stem Cells in Three-Dimensional Culture System

Kangli Cui[1,4] †, Yujuan Zhu[1,4] †, Yang Shi[5], Tingwei Chen[6], Hui Wang[1,4], Yaqiong Guo[1,4], Pengwei Deng[1,4], Haitao Liu[1,4], Xiaoguang Shao[5], and Jianhua Qin[1,2,3,4]*

† These authors contribute equally to this work.

[1] Division of Biotechnology, Dalian Institute of Chemical Physics, Chinese Academy of Sciences, Dalian 116023, China

[2] Institute for Stem Cell and Regeneration, Chinese Academy of Sciences, Beijing 100101, China

[3] CAS Center for Excellence in Brain Science and Intelligence Technology, Chinese Academy of Sciences, Shanghai 200031, China

[4] University of Chinese Academy of Sciences, Beijing 100049, China

[5] Dalian Municipal Women and Children's Medical Center, Dalian 116037, China

[6] Yunnan Key Laboratory of Primate Biomedical Research, Institute of Primate Translational Medicine, Kunming University of Science and Technology, Kunming, 650031, China.

* Correspondence should be addressed to J.Q. (jhqin@dicp.ac.cn).

Supporting information

Figures

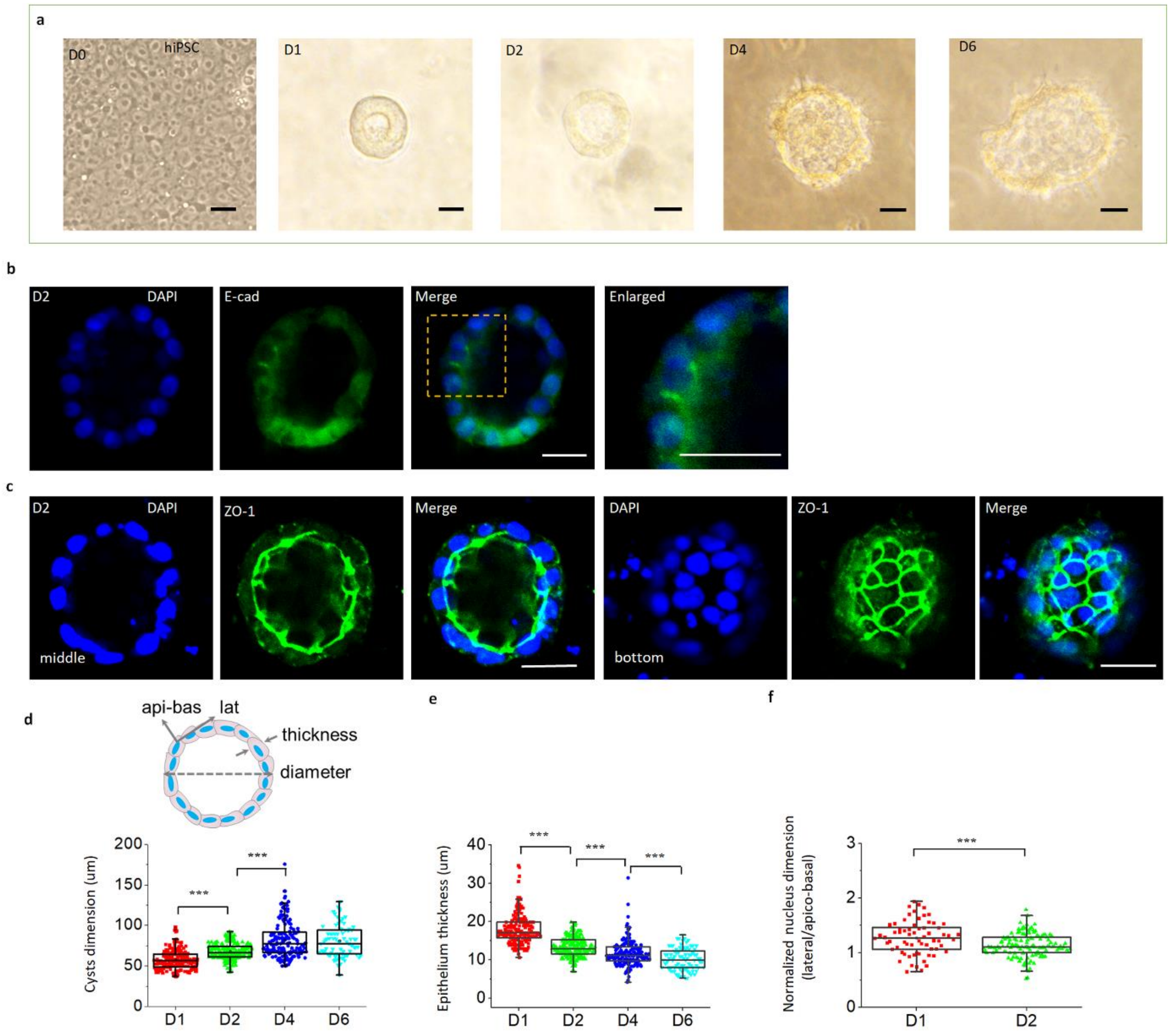

Figure. S1 hPSCs form blastocoel-like cavities in the Matrigel. a, Representative bright field images of squamous cavities during differentiation. **b,** Immunofluorescence staining of E-cad in cavities at day 2. **c,** Confocal micrographs taken at two planes passing the middle and bottom of the cavity are shown at day 2. Intact cavities were identified by the continuous expression of ZO-1 along the cell borders. Scale bars, 25 μm. **d,** Box charts showing cavity dimension in the Matrigel (box:

25%-75%, bar-in box: median, and wiskers: 1% and 99%). Ncavity = 92, 105, 87 and 45 for day 1, 2, 4 and 6, respectively. Student's *t*-test, * $p < 0.05$, ** $p < 0.01$, *** $p < 0.001$. **e,** Box charts showing epithelium thickness for hPSC cavities. Ncavity = 92, 105, 87 and 45 for day 1, 2, 4 and 6, respectively. **f,** Box charts showing the laterral (lat) and the apico-basal (api-bas) direction. Ncell = 66, 132 and Ncavity = 10, 10 for day 1 and 2, respectively.

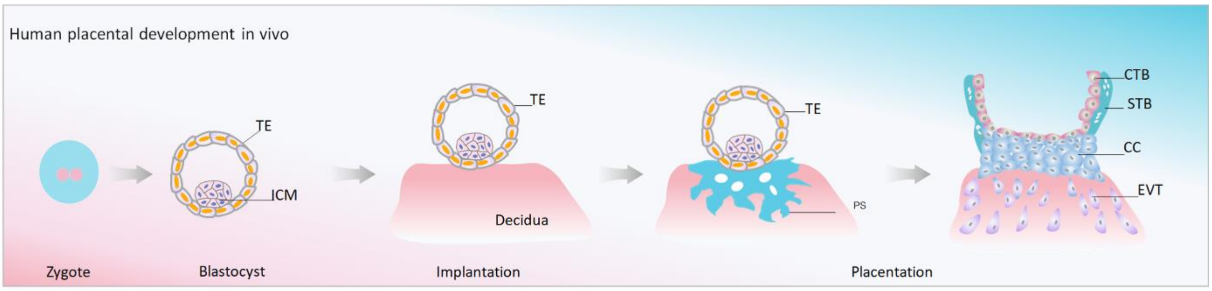

Figure. S2 Schematic of placental development in human embryo *in vivo*. Placentation initiates from dozens of trophectoderms (TEs), the outer layer of blastocyst. Once the embryo implanted, TE invades into maternal decidua, and gives rise to the primitive syncytium (PS) and proliferative cytotrophoblasts (CTBs). CTBs subsequently proliferate and differentiate into trophoblast subtypes including syncytiotrophoblasts (STBs) and invasive extravillous trophoblasts (EVTs), and finally develop into complex placental tree. The multilayered cell column is characterized with a proliferative cytotrophoblast cell column (CC) at the base. ICM, inner cell mass.

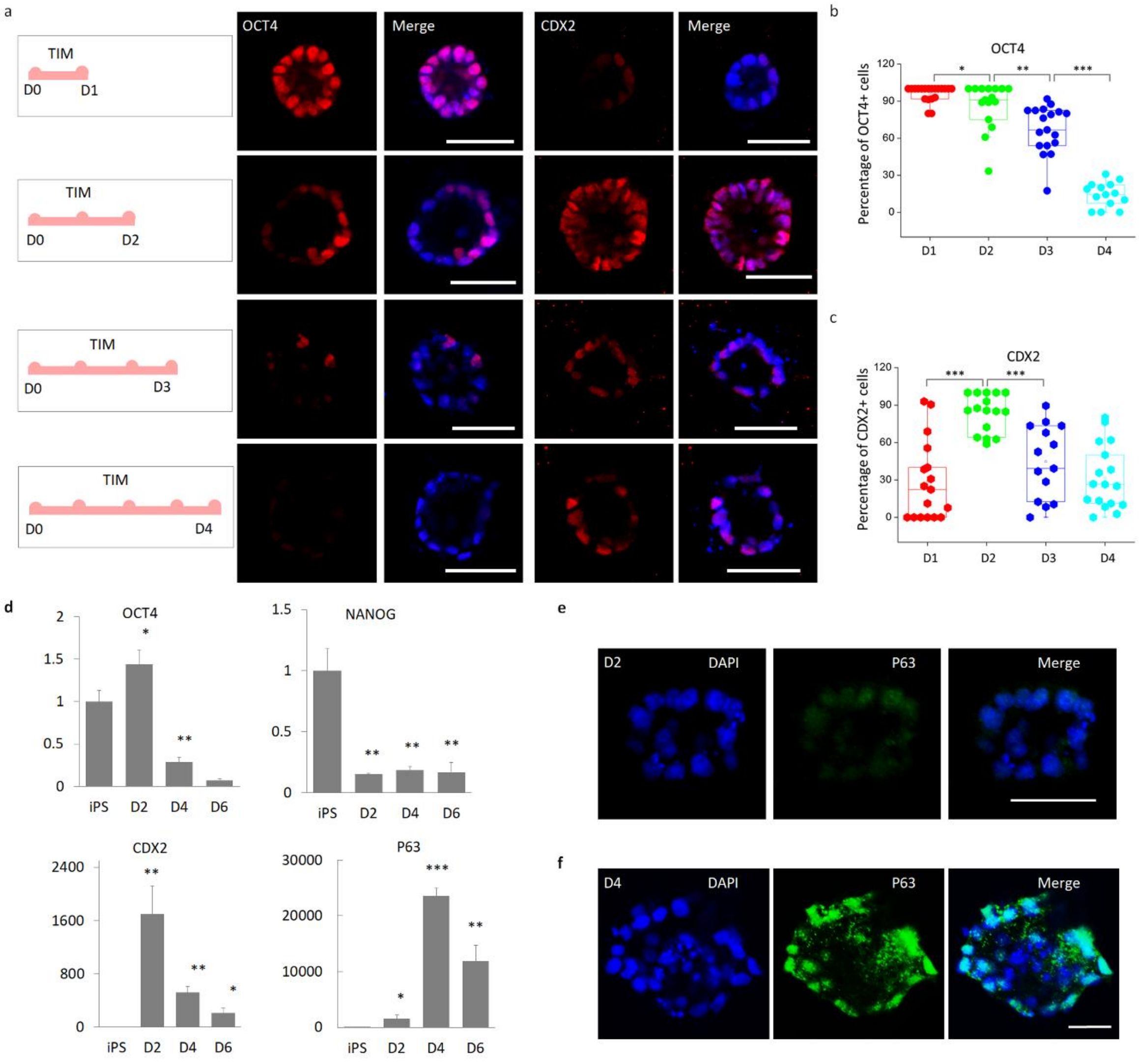

Figure. S3 Characterization of trophoblast precursor cells in 3D cavities from hPSCs. a, Confocal micrographs showing trophoblast precursor markers OCT4 and CDX2 in blastocoel-like cavity at day 1 to 4 with trophoblast introduction medium (TIM). Scale bars, 50 µm. **b,** Box charts showing percentage of OCT4+ cells in trophoblastic cavity (box: 25%-75%, bar-in box: median, and wiskers: 1% and 99%). N*cavity* = 20, 16, 18 and 14, N*cell* = 343, 296, 318 and 216 for day 1, 2, 3 and 4, respectively. **c,** Box charts showing percentage of CDX2+ cells in trophoblastic cavity (box: 25%-75%, bar-in box: median, and wiskers: 1% and 99%). N*cavity* = 17, 14, 16 and 17, N*cell* = 271, 286, 307 and 296 for day 1, 2, 3 and 4, respectively. Student's *t*-test, * $p < 0.05$, ** $p < 0.01$, *** $p < 0.001$. **d,** Analysis of qRT-PCR showing mRNA expression of pluripotent genes (NANOG, OCT4),

TE genes (OCT4, CDX2), and CTB genes (CDX2, P63). In contrast to the downregulation of the pluripotent gene NANOG, OCT4 was significantly upregulated with the treatment of BMP4 for 2 days. Consistently, the expression of CDX2 and P63 were greatly increased, but peaked at day 2 and day 4, respectively, implying the induction of trophoblast lineage in a sequential manner. The expression value was normalized to the GAPDH expression level. n = 3 replicates. Three independent experiments have been performed. Data are mean±SD. Student's *t*-test, * $p < 0.05$, ** $p < 0.01$, *** $p < 0.001$. **e-f,** Confocal micrographs showing P63 in cavities at day 2 and 4. The expression of P63 was very low or undetectable before day 4 with BMP4. Scale bars, 25 μm.

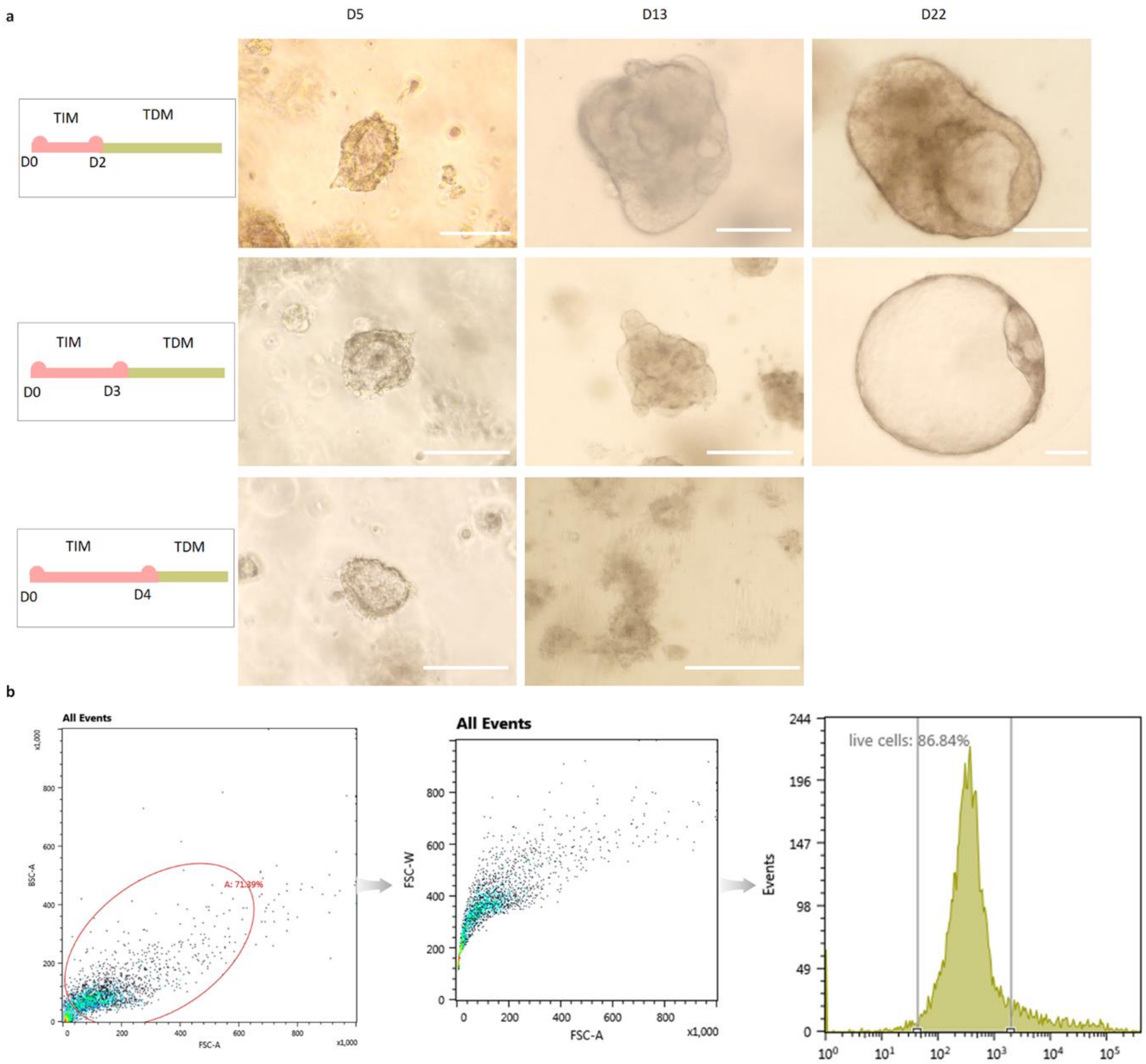

Figure. S4 Formation of trophoblast-like tissue from hPSCs. a, Representative phase contrast images of trophoblast-like tissue (TS tissue). Before culture in trophoblast differentiation medium (TDM), hPSCs were induced to trophoblastic lineage in TIM with BMP4 for 2, 3 or 4 days. In the presence of BMP4 for 2 and 3 days, hPSCs gradually self-organized into TS tissue, which contain cavity-like structure formed by STB. But with the treatment of BMP4 for a longer time (4 days), tissues failed to form TS tissue and finally collapsed. Scale bars, 100 μm. **b,** Flow cytometric analysis of single, live cells from TS tissue. A high proportion of live cells (~ 86.84%) was identified, indicating very limited cell death in TS tissue at 25 days of differentiation.

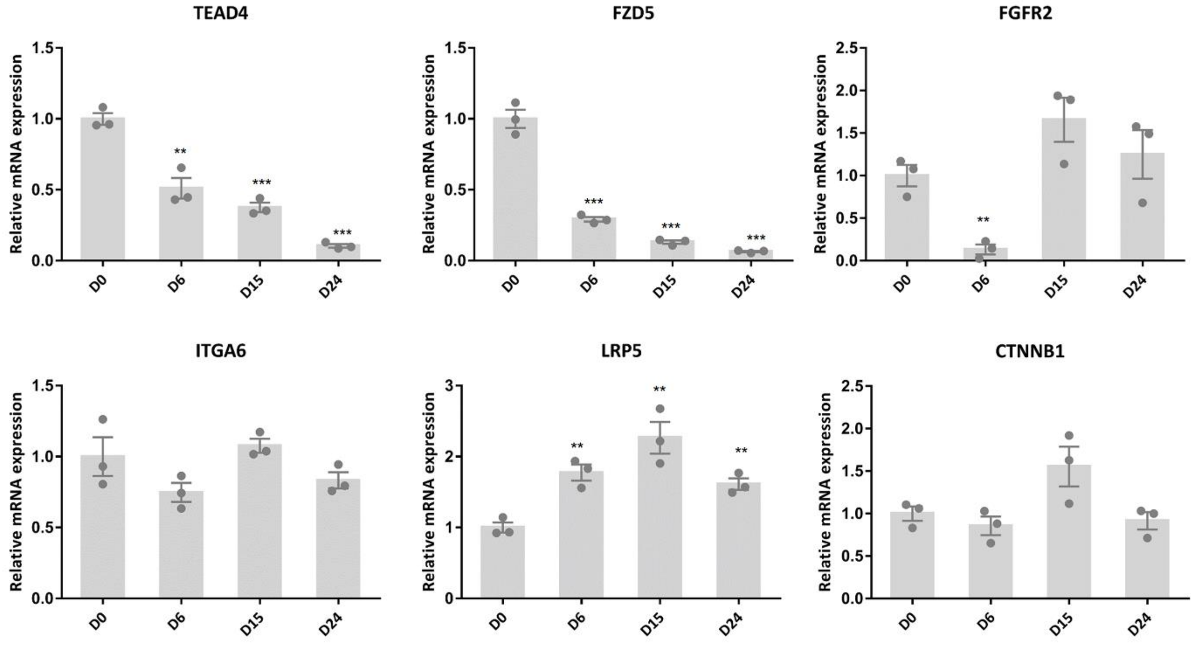

Figure. S5 Identification of CTB markers in trophoblast-like tissue. qPCR showing mRNA expression of CTB identity markers in trophoblast-like tissue (TS tissue) at 0, 6, 15 and 24 days of differentiation. hPSCs were set as the control group (D0). In contrast to most CTB markers detected in this study, TEAD4 and FZD5 show lower expression in 3D tissue compared to hPSCs. But similar expression pattern was also observed in first-trimester placenta (see **Figure. 4c**), implying the difference between trophoblast precursors and TS tissue. Three samples of each group were collected from two independent experiments. Data are mean±SEM. Student's *t*-test, * $p < 0.05$, ** $p < 0.01$, *** $p < 0.001$.

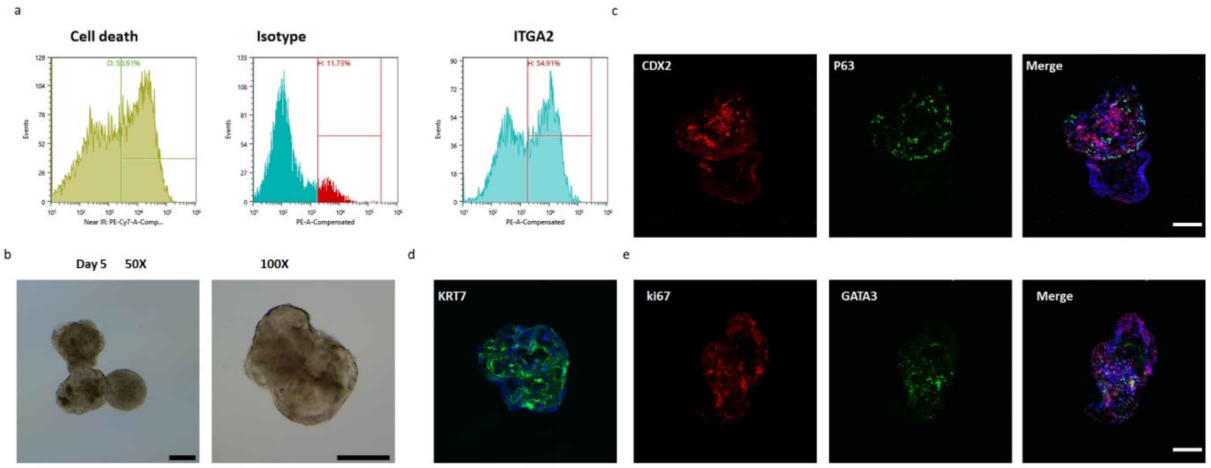

Figure. S6 Characterizing renewal ability of trophoblast-like tissue after freezing and thawing. **a,** Flow cytometric analysis of cell viability and trophoblast progenitor cells from trophoblast-like tissue (TS tissue) after thawing for 5 days. **b,** Representative bright field images of TS tissue at day 5 after thawing. Red asterisks indicate the characteristic cavity-like structure. Scale bar, 200 µm. **c-e**, Immunofluorescence staining of CTB markers CDX2, P63, KRT7, GATA3 and proliferative cell marker Ki67. Scale bar, 100 µm.

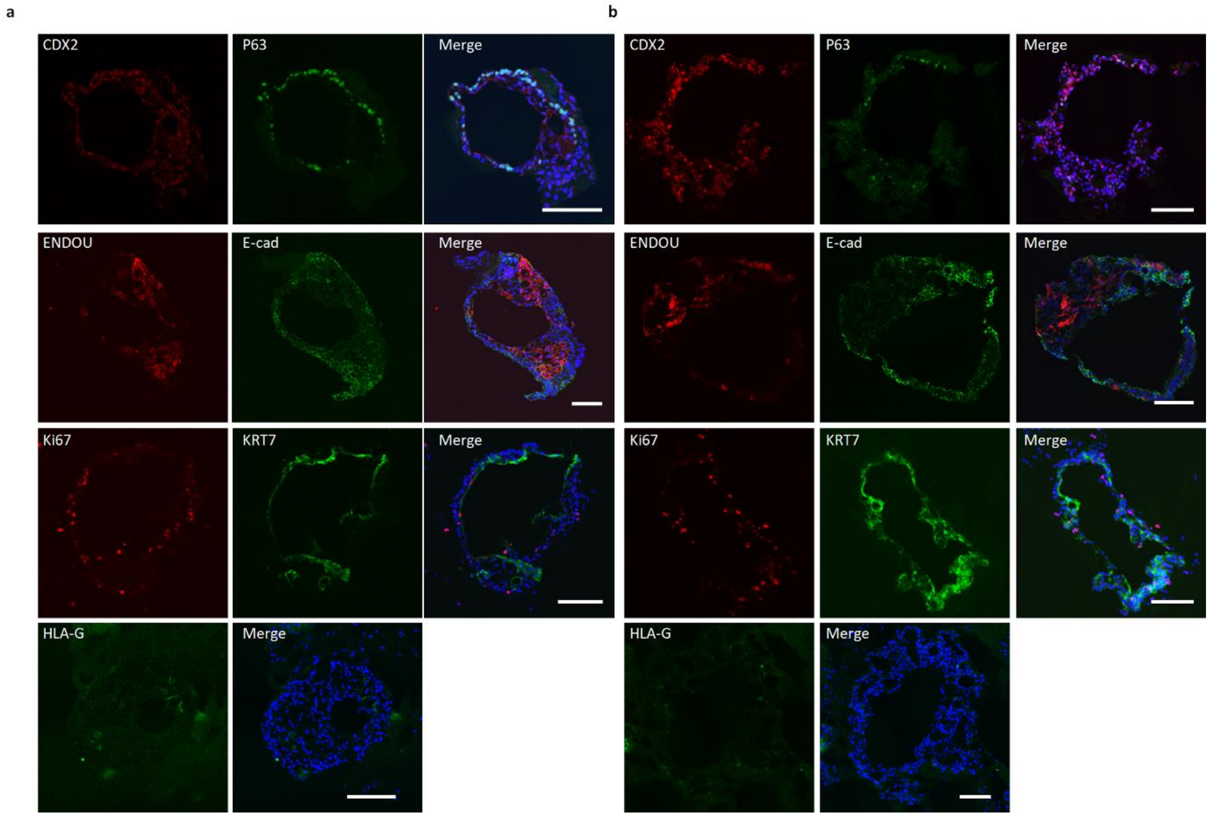

Figure. S7 Analysis of trophoblast-like tissue formation from hiPSC and hESC. Confocal micrographs showing the differentiation of CTB markers (P63, CDX2), trophoblast marker (KRT7), STB marker (ENDOU), EVT marker (HLA-G), and E-cad in trophoblast-like tissue (TS tissue) from hiPSC (day 33, **a**) and hESC (day 19, **b**). Scale bars, 100 μm.

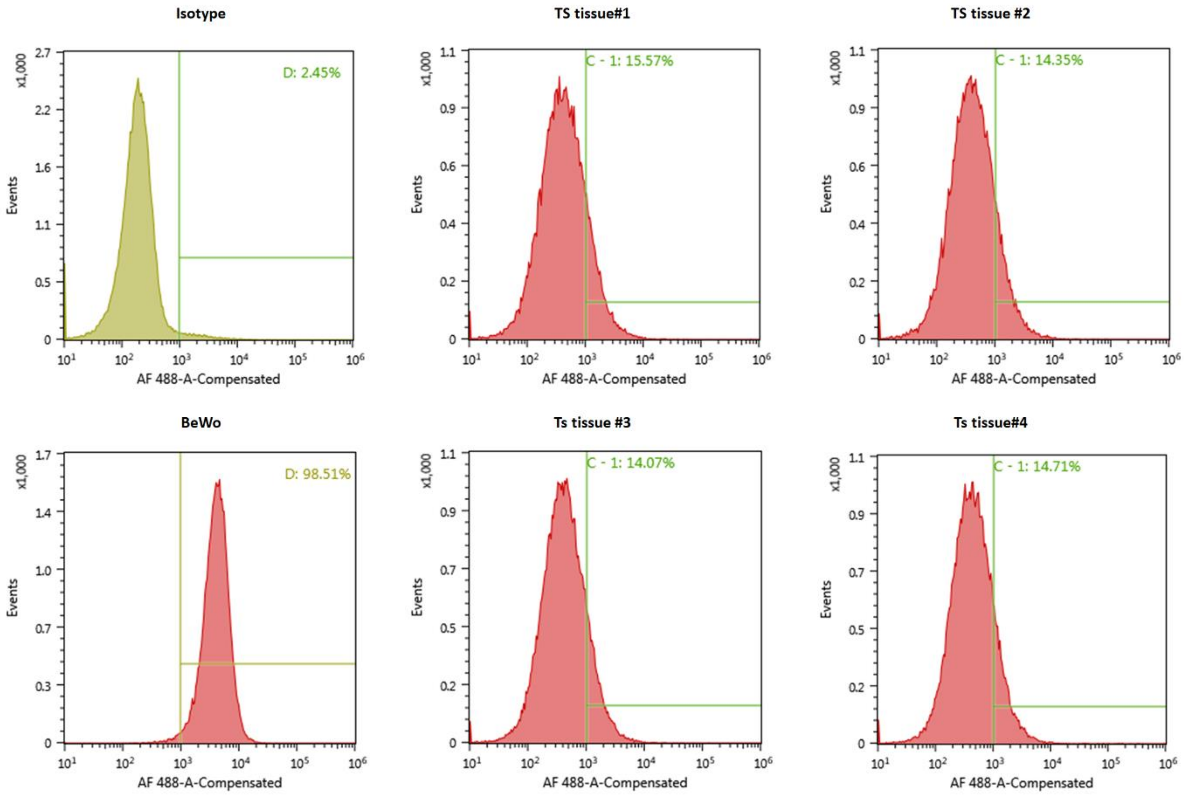

Figure. S8 FACS analysis of HLA class I molecules in trophoblast-like tissue. FACS analysis of four trophoblast-like tissue (TS tissue#1-4) and BeWo cells (positive control) with the Alexa-488-conjugated monoclonal antibody clone W6/32, which binds all HLA class I molecules. The experiments have been performed twice.

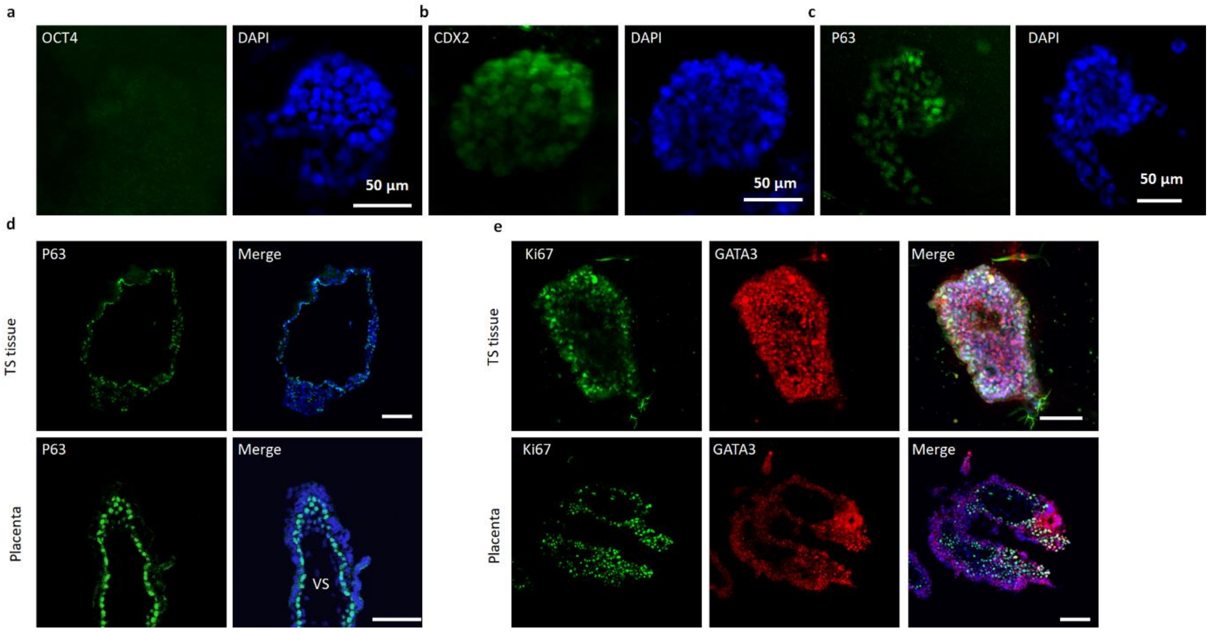

Figure. S9 Identification of trophoblast precursors in trophoblast-like tissue. a-c, Confocal micrographs showing trophoblast precursor markers OCT4, CDX2 and P63 at 6 days of differentiation. With the differentiation of CDX2+/P63+ trophoblasts, the expression of OCT4 was lost. **d-e,** Confocal micrographs showing the differentiation of trophoblast markers P63 and GATA3 in 3D tissue at 26 and 18 days of differentiation and first-trimester placenta (Placenta) (n = 3; 6–8 weeks gestation). The characterized cavity-like structure of STB was formed in trophoblast-like tissue (TS tissue). The expression of Ki67 revealed the proliferative cells. Scale bars, 50 µm (a-c), 100 µm (d and e, the upper), 200 µm (e, the bottom).

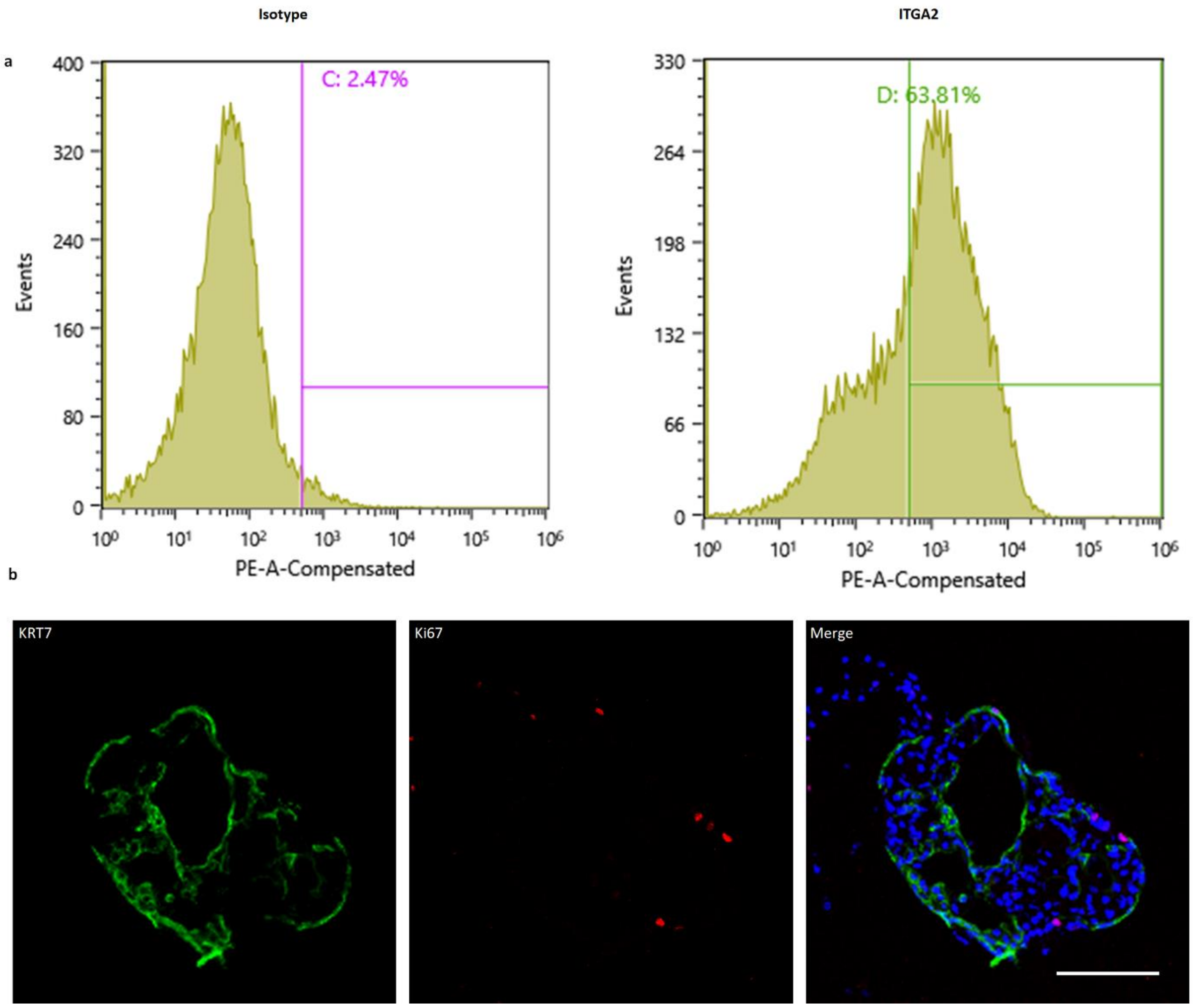

Figure. S10 Identification of trophoblast precursors in trophoblast-like tissue. a, FACS analysis of ITGA2 in trophoblast-like tissue (TS tissue) at 25 days of differentiation. ITGA2+ cells gradually decreased to ~ 60%. **b,** Confocal micrographs showing proliferative Ki67+ cells and KRT7+ trophoblasts from 33 days of differentiation. With the differentiation, proliferative cells were down regulated. Scale bars, 100 μm.

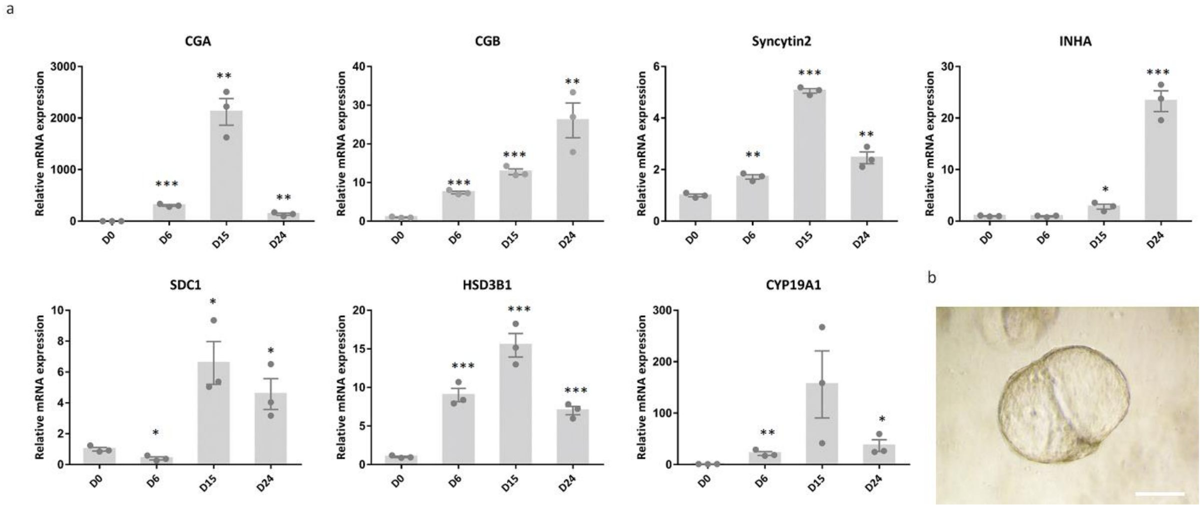

Figure. S11 Identification of trophoblast subtype STB in trophoblast-like tissue. a, qPCR showing mRNA expression of STB identity markers in trophoblast-like tissue (TS tissue) at 0, 6, 15 and 24 days of differentiation. hPSCs were set as the control group (D 0). Syncytial genes are expressed at higher levels with the growth of TS tissue, indicating the differentiation toward STB. Three samples of each group were collected from two independent experiments. Data are mean±SEM. Student's *t*-test, * $p < 0.05$, ** $p < 0.01$, *** $p < 0.001$. **b,** Representative contrast image of cavity-like structure formed by STB at 17 days of differentiation. Scale bars, 200 μm.

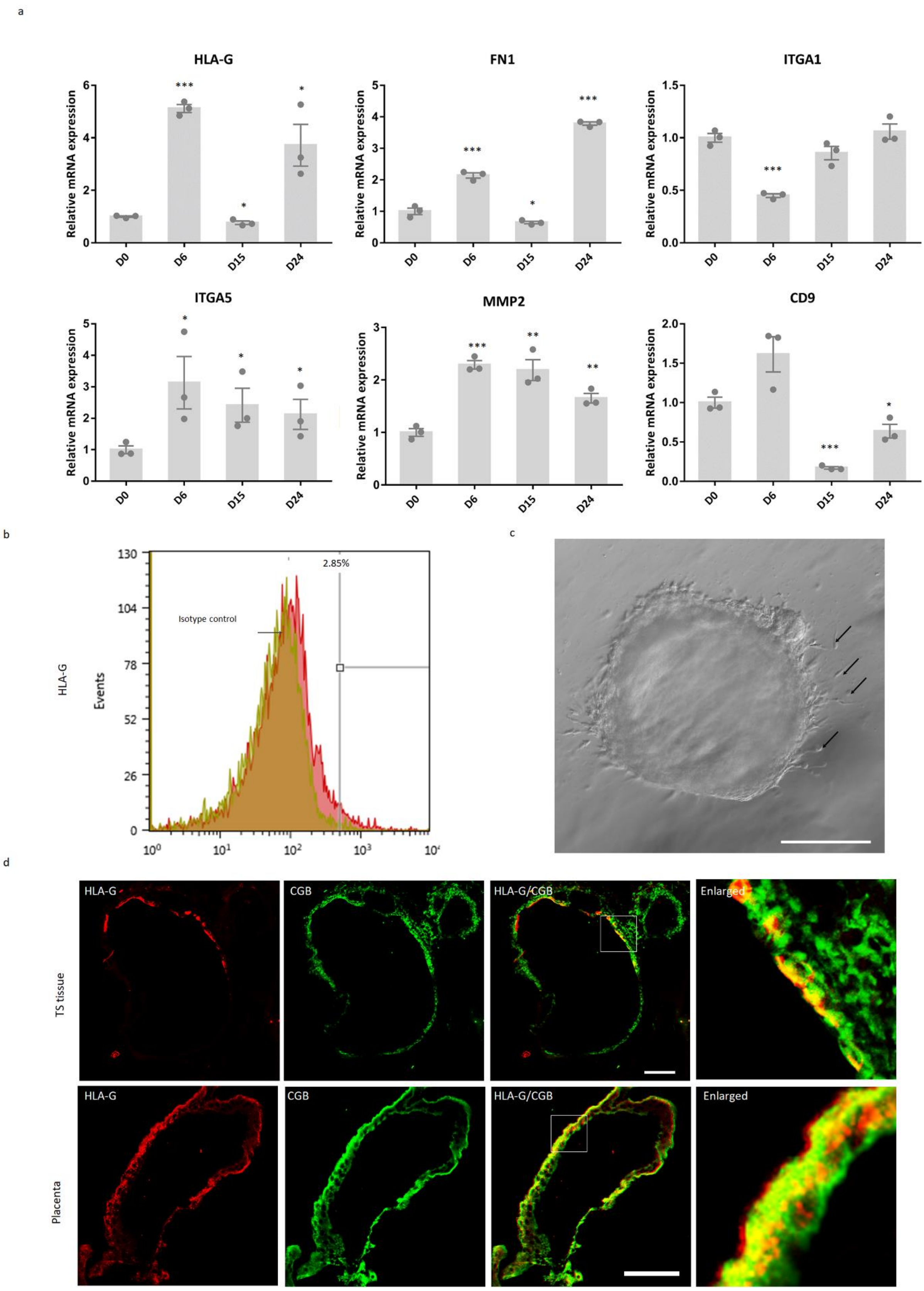

Figure. S12 Identification of trophoblast subtype EVT in trophoblast-like tissue. a, qPCR showing mRNA expression of EVT markers in trophoblast-like tissue (TS tissue) at 0, 6, 15 and 24

days of differentiation. hPSCs were set as the control group (D 0). All EVT genes show much higher expression levels in comparison to hPSCs, although CD9 shows lower expression in TS tissue. Three samples of each group were collected from two independent experiments. Data are mean±SEM. Student's *t*-test, * $p < 0.05$, ** $p < 0.01$, *** $p < 0.001$. **b,** Flow cytometric analysis revealed a relative low level of HLA-G+ EVT (~ 3%) in TS tissue. **c,** Characterization of EVT migration in TS tissue. The arrow indicated the migrated EVT. Scale bar, 200 μm. **d,** Immunofluorescence images showing HLA-G+ EVTs and CGB+ STBs in TS tissue at day 33 and first-trimester placenta (Placenta) (n = 3; 6–8 weeks gestation). Scale bars, 100 μm.

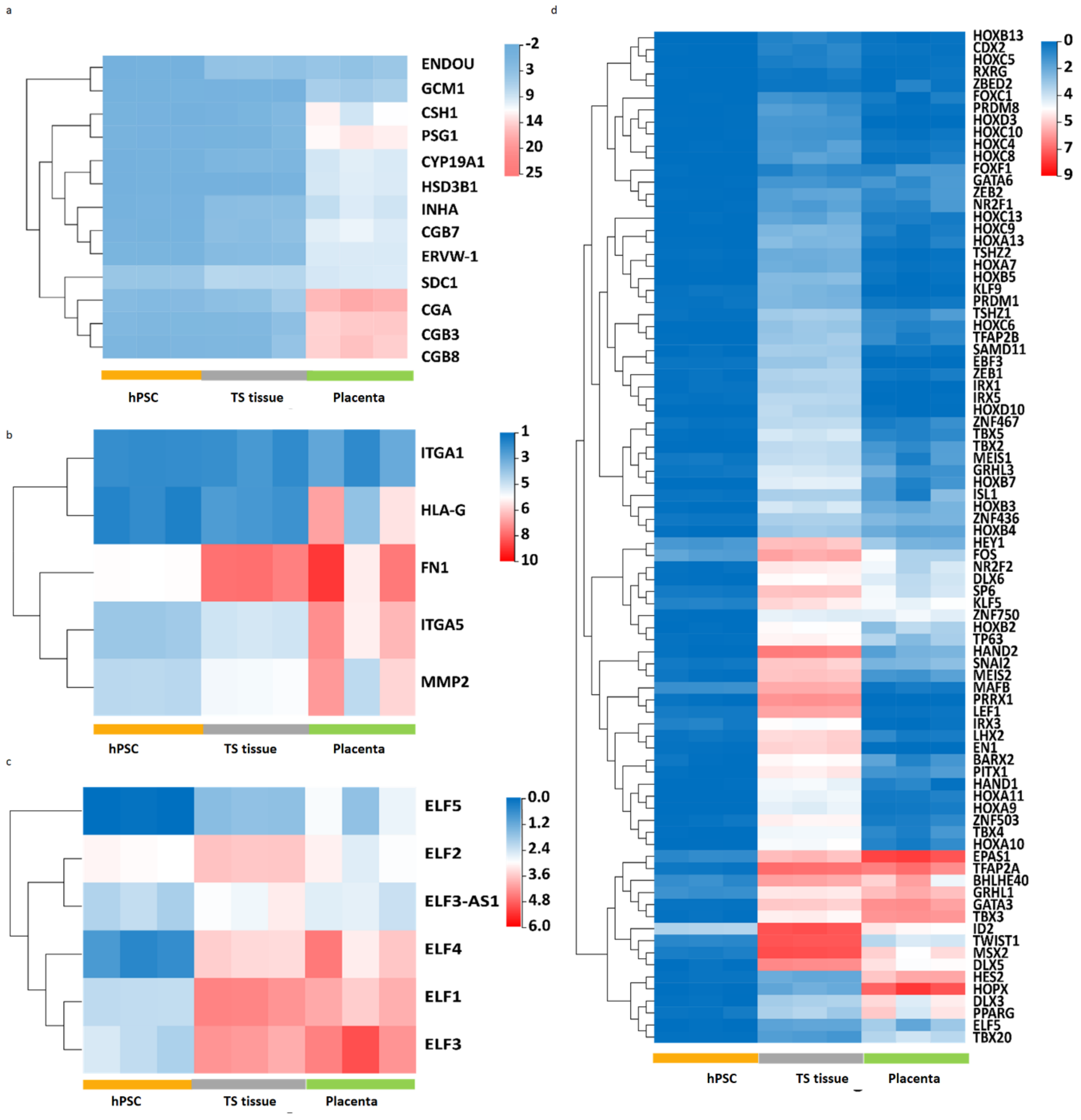

Figure. S13 Bioinformatic analysis of trophoblast-like tissue and primary placental villi. a-b, Clustered heatmap depicting selected expression patterns of markers of STB and EVT. In trophoblast-like tissue (TS tissue) at day 24, syncytial genes show the moderate upregulation with a few exceptions (e.g., GCM1, HSD3B1), but very low or no expression in hPSC (**a**). EVT markers exhibited similar expression patterns in 3D tissue and placenta, such as FN1, ITGA5 and MMP2 (**b**). These alterations are in the agreement with qPCR and immunofluorescence data. **c,** Heat map of genes from the ELF family of transcription factors. ELF1 and ELF3 are similar in all samples, but higher

in placenta and 3D tissue in comparison to hPSCs. ELF4 and ELF5 show significant higher expression levels in placenta and TS tissue. **d,** Heat map highlighting transcription factors from the differentially expressed genes between TS tissue, Placenta, and hPSC.

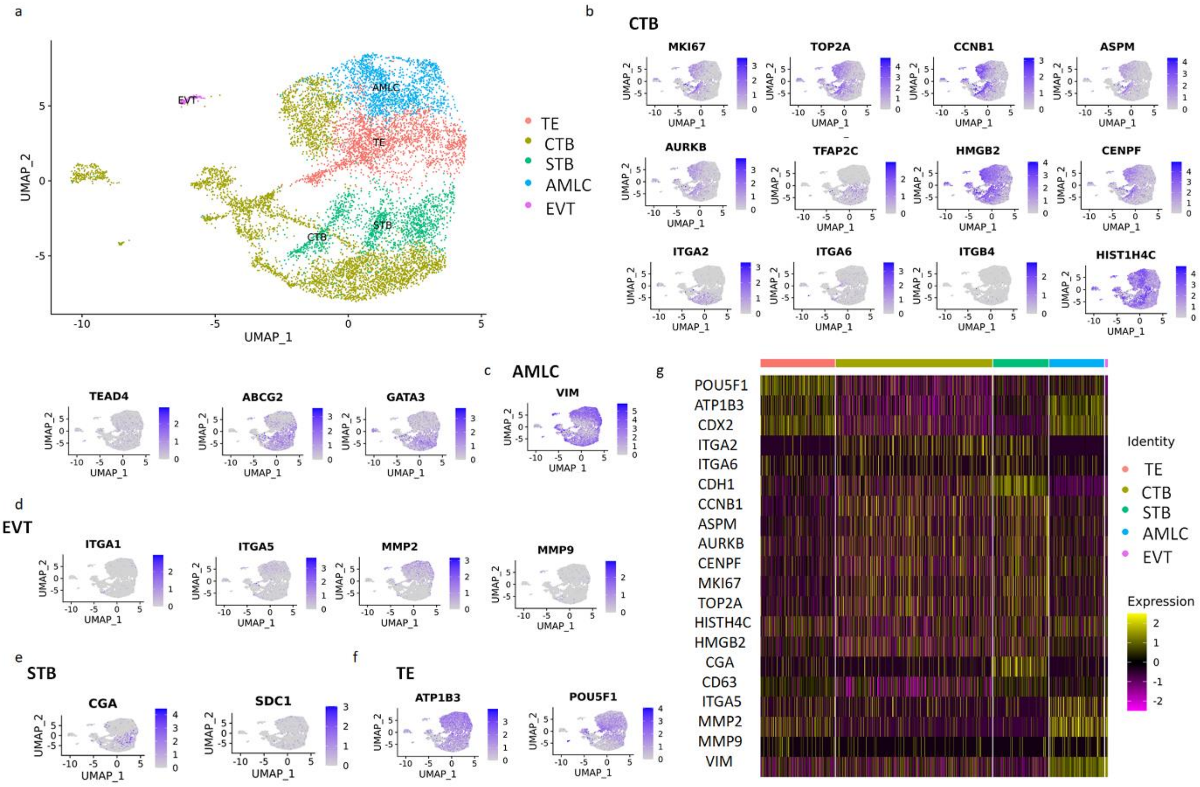

Figure. S14 Analysis of trophoblast-like tissue at 6 days of differentiation. **a,** UMAP plot displaying 10225 cells from 10x Genomics scRNA-seq analysis after 6 days of differentiation. Unsupervised clustering identified 5 major clusters that are marked by different colors. **b-f,** Feature plot of different cell types (CTB, AMLC, EVT, STB, and TE) in the different clusters. **g,** Heat map of differentially expressed genes in different clusters.

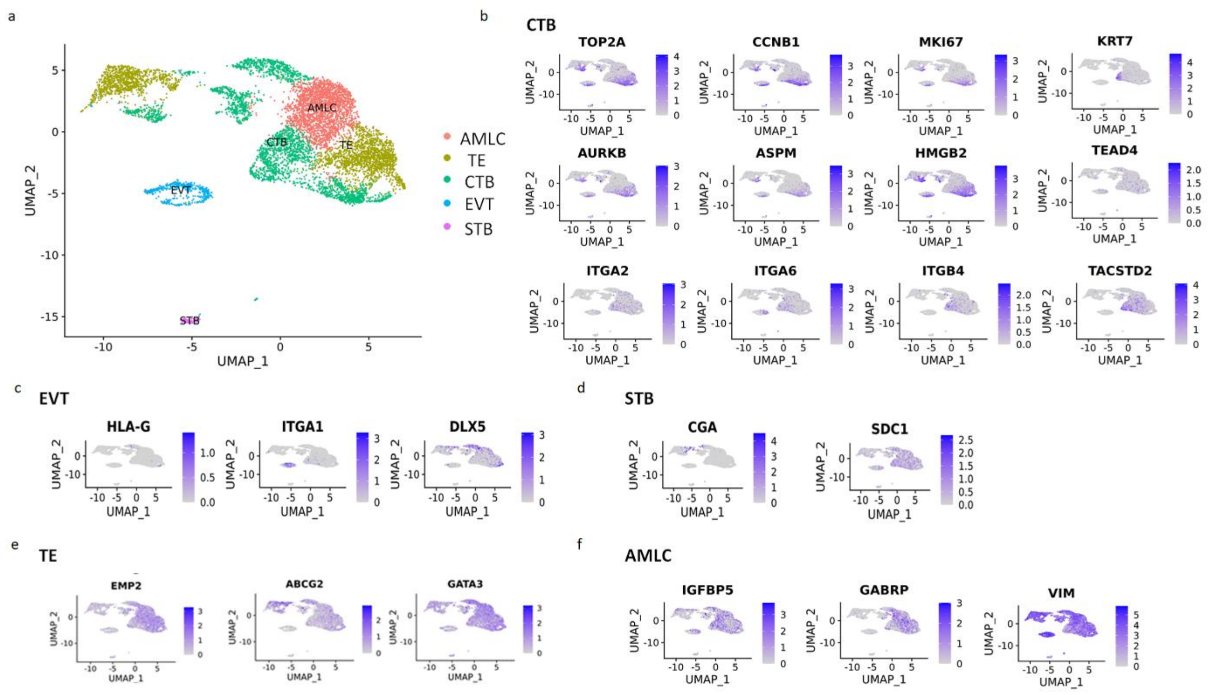

Figure. S15 Analysis of trophoblast-like tissue at 12 days of differentiation. **a,** UMAP plot displaying 8616 cells from 10x Genomics scRNA-seq analysis after 12 days of differentiation. **b-f,** Feature plot of different cell types (CTB, EVT, STB, TE and AMLC) in the different clusters.

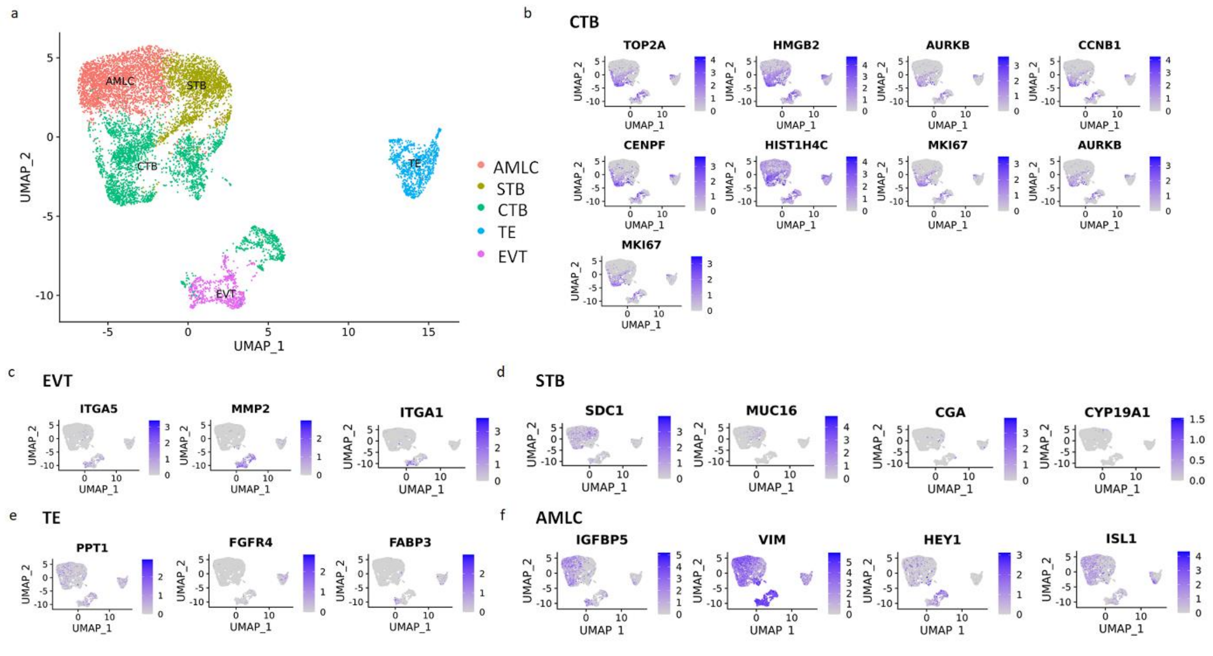

Figure. S16 Analysis of trophoblast-like tissue at 18 days of differentiation. **a,** UMAP plot displaying

9850 cells from 10x Genomics scRNA-seq analysis after 18 days of differentiation. **b-f,** Feature plot of different cell types (CTB, EVT, STB, TE, and AMLC) in the different clusters.

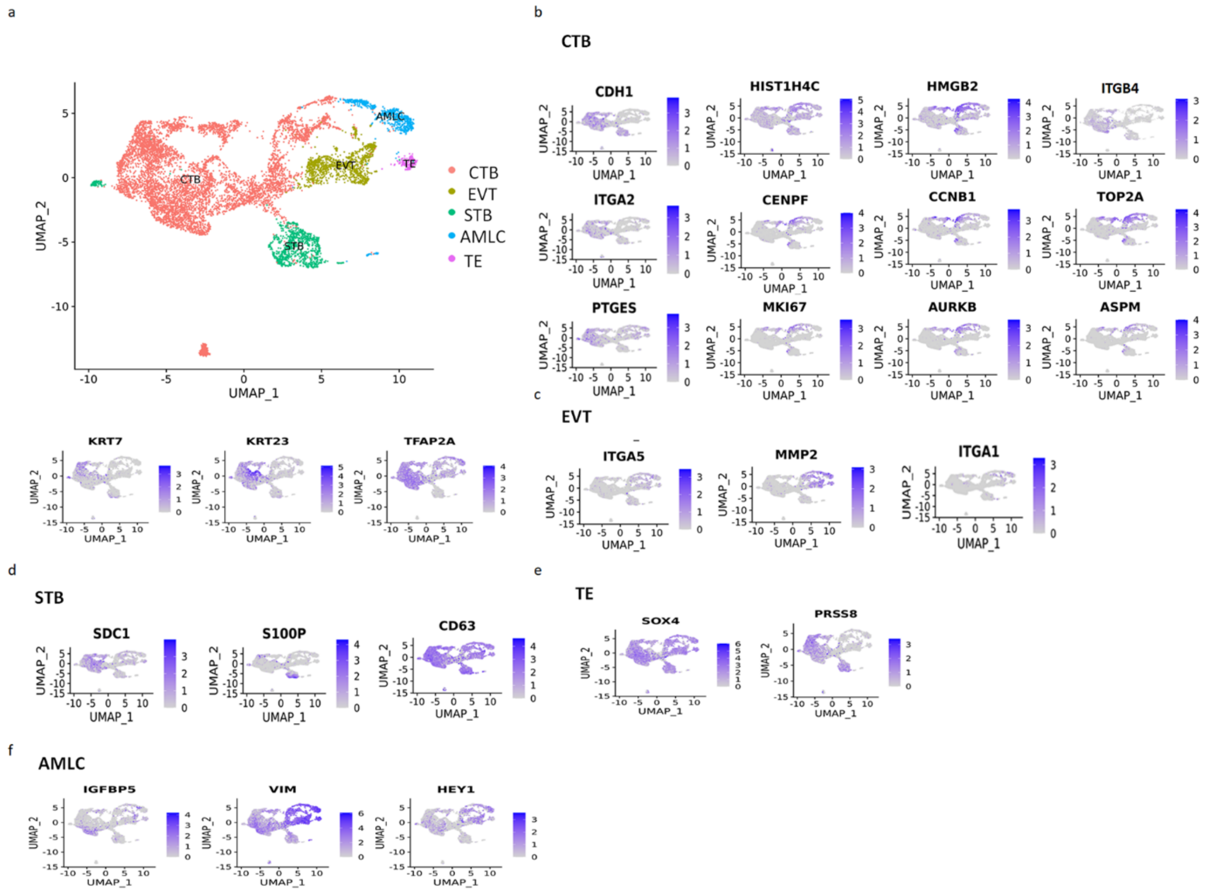

Figure. S17 Analysis of trophoblast-like tissue at 27 days of differentiation. **a,** UMAP plot displaying 9381 cells from 10x Genomics scRNA-seq analysis after 27 days of differentiation. The cell number of CTBs, STB, EVT, TE, and AMLC were 6448, 954, 1144, 173, and 662 respectively. CTB, cytotrophoblast; STB, syncytiotrophoblast; EVT, extravillous trophoblast. **b-f,** Feature plot of different cell types (CTB, EVT, STB, TE, and AMLC) in the different clusters.

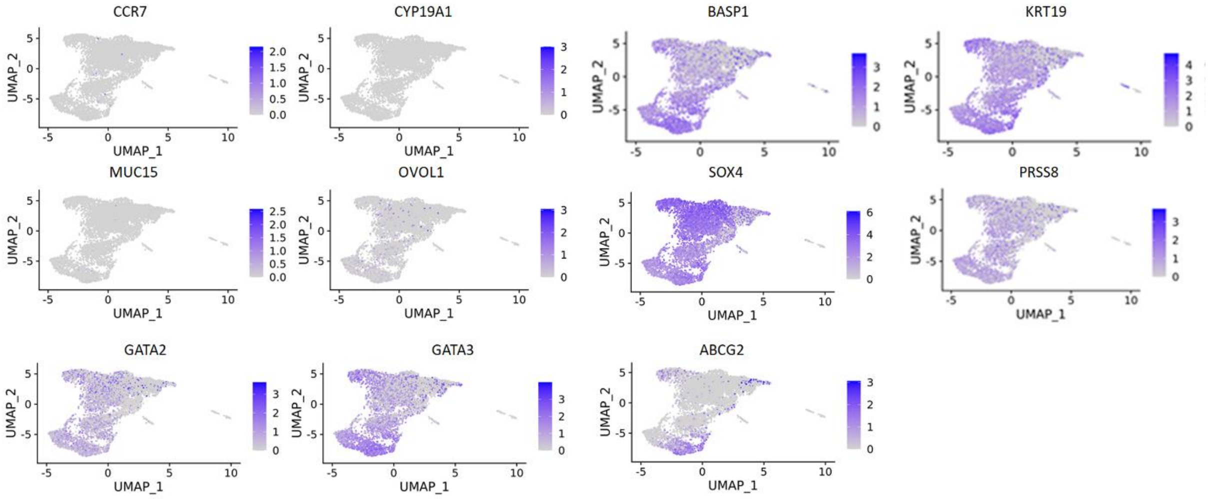

Figure. S18 UMAP feature plot of trophectoderm-specific gene in the formed tissue at 2 days of differentiation. CCR7, MUC15, CYP19A1, and OVOL1 were referred as polar trophectoderm (TE)-specific gene.

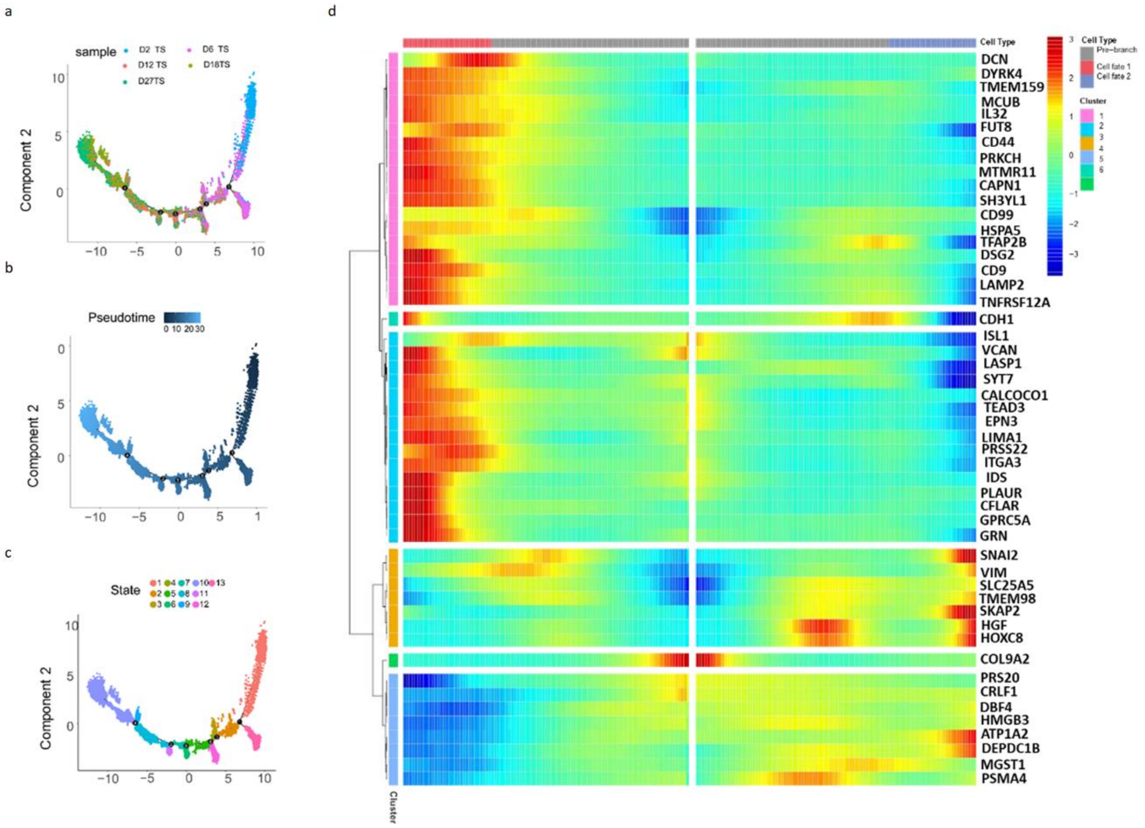

Figure. S19 Unsupervised lineage trajectory analysis of trophoblast-like tissue. **a,** Lineage trajectory of the cell subcluster by samples. **b-c,** Lineage trajectory of the cell subcluster by pseudotime (**b**), or by cell state (**c**). **d,** Heatmap of branched expression of top50 DEGs at point 1, as indicated in panel **c**.
