# Supplementary material for: Establishment of Trophoblast‐Like Tissue Model from Human Pluripotent Stem Cells in Three‐Dimensional Culture System
Source: Adv Sci (Weinh). 2021 Nov 23;9(3):2100031. doi: 10.1002/advs.202100031 (PMC8787386; doi:10.1002/advs.202100031)
Supplement: Supplementary file 2 — Supplemental Table 1 [file ADVS-9-2100031-s006.pdf]

Primer pairs used to examine mRNA expression.

Primer name	Sequence
OCT4	Fw 5′-GGA GAA GCT GGA GCA AAA CC-3′<br>Rv 5′-TGG CTG AAT ACC TTC CCA AA-3′
TEAD4	Fw 5′-CAG GTG GTG GAG AAA GTT GAG A-3′<br>Rv 5′-GTG CTT GAG CTT GTG GAT GAA G-3′
ELF5	Fw 5′-AGT CTG CAC TGA CAT TTT CTC ATC-3′<br>Rv 5′-CAG AAG TCC TAG GGG CAG TC-3′
EOMES	Fw 5′-CCC TGG AGG TCG GTA CGG CG-3′<br>Rv 5′-ACA AGC CAC CGC TGG GGA GG-3′
KRT7	Fw 5′-TGA ATG ATG AGA TCA ACT TCC TCA G-3′<br>Rv 5′-TGT CGG AGA TCT GGG ACT GC-3′
P63	Fw 5′-CTG GAA AAC AAT GCC CAG A-3′<br>Rv 5′-AGA GAG CAT CGA AGG TGG AG-3′
FZD5	Fw 5′-TTC TGG ATA GGC CTG TGG TC-3′<br>Rv 5′-AGG TAG CAG GCT GAC AGG AA-3′
ITGA6	Fw 5′-CAC ATC TCC TCC CTG AGC AC-3′<br>Rv 5′-TAT CTT GCC ACC CAT CCT TG-3′
LRP5	Fw 5′-AAA CAG CAG TGC GAC TCC TTC-3′<br>Rv 5′-TGG CAC ACA AAA TAG ACA CCA-3′
CTNNB1	Fw 5′-ATT TGA TGG AGT TGG ACA TGG C-3′<br>Rv 5′-CCA GCT ACT TGT TCT TGA GTG AAG-3′
SDC1	Fw 5′-CTA TTC CCA CGT CTC CAG AAC C-3′<br>Rv 5′-GGA CTA CAG CCT CTC CCT CCT T-3′
MMP2	Fw 5′-GTA TTT GAT GGC ATC GCT CA-3′<br>Rv 5′-CAT TCC CTG CAA AGA ACA CA-3′
GATA3	Fw 5′-GCC CCT CAT TAA GCC CAA G-3′<br>Rv 5′-TTG TGG TGG TCT GAC AGT TCG-3′
KRT17	Fw 5′-AAG ATC CGT GAC TGG TAC CAG AGG-3′<br>Rv 5′-GAT GTC GGC CTC CAC ACT CAG G-3′
Syncytin2	Fw 5′-CCT TCA CTA GCA GCC TAC CG-3′<br>Rv 5′-GCT GTC CCT GGT GTT TCA GT-3′
CGB	Fw 5′-CCC CTT GAC CTG TGA TGA CC-3′<br>Rv 5′-TAT TGT GGG AGG ATC GGG GT-3′
CGA	Fw 5′-ACATCCTGCAAAAAGCCCAGAGAAA-3′<br>Rv 5′-ACTGAAGTATTGGGGCACCCGG-3′
HLA-G	Fw 5′-GAG GAG ACA CGG AAC ACC AAG-3′<br>Rv 5′-GTC GCA GCC AAT CAT CCA CT-3′
CDX2	Fw 5′-GAC GTG AGC ATG TAC CCT AGC-3′<br>Rv 5′-GCG TAG CCA TTC CAG TCC T-3′
GAPDH	Fw 5′-GTG GAC CTG ACC TGC CGT CT-3′<br>Rv 5′-GGA GGA GTG GGT GTC GCT GT-3′
FGFR2	Fw 5′-GGTCGTTTCATCTGCCTGGT-3′<br>Rv 5′-CCTTCCCGTTTTTCAGCCAC-3′
HSD3B1	Fw 5′-AGAAGAGCCTCTGGAAAACACATG-3′

	Rv 5′-TAAGGCACAAGTGTACAGGGTGC-3′
CYP19A1	Fw 5′-TGCAAAGCACCCTAATGTTG-3′<br>Rv 5′-TTTGTCCCCTTTTTCACTGG-3′
INHA	Fw 5′-GTCTCCCAAGCCATCCTTTT-3′<br>Rv 5′-AGAGCTATTGGAGGCTGCTG-3′
FN1	Fw 5′-GAGGGACCTGGAAGTTGTTG-3′<br>Rv 5′-GCACAGTGAACTCCTGGACA-3′
ITGA5	Fw 5′-CCCCGAGTACCTGATCAAC-3′<br>Rv 5′-AGGGATCGAATGTCTGAGCC-3′
CD9	Fw 5′-GCATTGCCGTGGTCATGAT-3′<br>Rv 5′-TGCGGATAGCACAGCACAAG-3′
ITGA1	Fw 5′-CAAACTGCAGACCCCATATC-3′<br>Rv 5′-TGACTTGGCTGATGTCAGAA-3′
